# Decentralized Biobanking Apps for Patient Tracking of Biospecimen Research: Real-World Usability and Feasibility Study

**DOI:** 10.2196/70463

**Published:** 2025-04-10

**Authors:** William Sanchez, Ananya Dewan, Eve Budd, M Eifler, Robert C Miller, Jeffery Kahn, Mario Macis, Marielle Gross

**Affiliations:** 1 de-bi, co. Greencastle, PA United States; 2 Johns Hopkins School of Medicine Johns Hopkins University Baltimore, MD United States; 3 Harpur College of Arts and Sciences State University of New York Binghamton, NY United States; 4 Faculty of Medicine Mayo Clinic Rochester, MN United States; 5 School of Medicine Indiana University Hospital Indianapolis, IN United States; 6 Johns Hopkins Berman Institute of Bioethics Johns Hopkins University Baltimore, MD United States; 7 Carey School of Business Johns Hopkins University Baltimore, MD United States

**Keywords:** patient empowerment, biobanking, biospecimens, transparency, community engagement, nonfungible tokens, NFTs, blockchain technology, decentralized biobanking, pilot studies, technical feasibility, biowallet

## Abstract

**Background:**

Biobank privacy policies strip patient identifiers from donated specimens, undermining transparency, utility, and value for patients, scientists, and society. We are advancing decentralized biobanking apps that reconnect patients with biospecimens and facilitate engagement through a privacy-preserving nonfungible token (NFT) digital twin framework. The decentralized biobanking platform was first piloted for breast cancer biobank members.

**Objective:**

This study aimed to demonstrate the technical feasibility of (1) patient-friendly biobanking apps, (2) integration with institutional biobanks, and (3) establishing the foundation of an NFT digital twin framework for decentralized biobanking.

**Methods:**

We designed, developed, and deployed a decentralized biobanking mobile app for a feasibility pilot from 2021 to 2023 in the setting of a breast cancer biobank at a National Cancer Institute comprehensive cancer center. The Flutter app was integrated with the biobank’s laboratory information management systems via an institutional review board–approved mechanism leveraging authorized, secure devices and anonymous ID codes and complemented with a nontransferable ERC-721 NFT representing the *soul-bound* connection between an individual and their specimens. Biowallet NFTs were held within a custodial wallet, whereas the user experiences simulated token-gated access to personalized feedback about collection and use of individual and collective deidentified specimens. Quantified app user journeys and NFT deployment data demonstrate technical feasibility complemented with design workshop feedback.

**Results:**

The decentralized biobanking app incorporated key features: “biobank” (learn about biobanking), “biowallet” (track personal biospecimens), “labs” (follow research), and “profile” (share data and preferences). In total, 405 pilot participants downloaded the app, including 361 (89.1%) biobank members. A total of 4 central user journeys were captured. First, all app users were oriented to the ≥60,000-biospecimen collection, and 37.8% (153/405) completed research profiles, collectively enhancing annotations for 760 unused specimens. NFTs were minted for 94.6% (140/148) of app users with specimens at an average cost of US $4.51 (SD US $2.54; range US $1.84-$11.23) per token, projected to US $17,769.40 (SD US $159.52; range US $7265.62-$44,229.27) for the biobank population. In total, 89.3% (125/140) of the users successfully claimed NFTs during the pilot, thereby tracking 1812 personal specimens, including 202 (11.2%) distributed under 42 unique research protocols. Participants embraced the opportunity for direct feedback, community engagement, and potential health benefits, although user onboarding requires further refinement.

**Conclusions:**

Decentralized biobanking apps demonstrate technical feasibility for empowering patients to track donated biospecimens via integration with institutional biobank infrastructure. Our pilot reveals potential to accelerate biomedical research through patient engagement; however, further development is needed to optimize the accessibility, efficiency, and scalability of platform design and blockchain elements, as well as a robust incentive and governance structure for decentralized biobanking.

## Introduction

### Background

University biobanks collect, store, and distribute biospecimens such as tissue and blood, capitalizing on leftover clinical materials from affiliated hospitals to drive biomedical science and drug discovery [1-3]. Standard operating procedure for most biobanks in academic medical centers includes prospective broad consent for nonspecific, future research [4] coupled with deidentification, whereby identifiers are stripped before specimen allocation [5]. In this setting, patients do not learn what becomes of their donations, and scientists lack access to the donor, linked specimens, and evolving clinical data [4,6]. This disconnect, though the by-product of policies designed to protect privacy while promoting learning, promulgates a biobank ecosystem that permits problematic gaps in recognition, reciprocity, and return of results [7,8]. Simultaneously, vast yet siloed specimen collections have accumulated across most US academic medical centers, a widely underused and unsustainable “treasure trove” wherein frozen assets lay hidden from patients and scientists for whom they may be most valuable [3,9]. The lack of an efficient market for ensuring the use of donated materials deepens the crisis of faith in public health institutions and has prompted attempts at marketplace solutions [10,11].

We are advancing *decentralized biobanking* as a software platform predicated on blockchain technology’s democratic ethos, incentive alignment, transparency, and assurances of trust [12]. These key features are reflective of blockchains as permissionless, distributed, shared ledgers of digital transactions engineered to be mathematically concordant, accessible, and auditable [13], underscoring their first and most successful use to date for the creation of global digital currency such as Bitcoin, which makes them fit for purpose in efforts to decentralize ownership and governance of data through thoughtfully structured peer-to-peer networks [14]. One of the most promising innovations enabled by blockchains are nonfungible tokens (NFTs), digital record identifiers that serve as electronic deeds for provably unique digital or physical assets that may be represented “on-chain” [15]. The potential for blockchain and NFTs to play a role in restructuring control and ownership of data has been widely discussed, with several notable projects in the health care domain [16,17]. Although empowering patient ownership of health data is compelling in theory, full realization of such initiatives has been elusive in light of complex regulatory considerations, socioeconomic factors, and technical limitations for blockchain technologies and legacy systems [18,19].

Building on the success and diversity of blockchain applications for decentralized finance [20,21], decentralized biobanking applies human-centered design and innovative system mechanisms to empower patients to track donated biospecimens and engage in downstream research activities, outcomes, and products via a platform compatible with established privacy policies and workflows. Our approach provides patients with secure, direct access to personal specimen data housed in institutional databases via user-friendly mobile and web apps complemented with a privacy-preserving NFT digital twin framework [22]. This strategy may support stepwise adoption of increasingly autonomous and progressively decentralized collaborations among patients, scientists, and physicians in a dynamic biomedical metaverse, or “biomediverse.”

### Objectives

Successful implementation of decentralized biobanking will usher in a new standard for research transparency, foster institutional accountability to the patients and communities they serve, and create opportunities to unite siloed datasets, facilitate timely translation of precision medicine and enable structurally just marketplace solutions for improving efficiency and effectiveness in the management of one of our most precious human resources. In this paper, we explore the technical feasibility of decentralized biobanking through a description and quantitative analysis of a live pilot for a breast cancer biobank at a US academic medical center. We discuss system design, key features, and NFT functionality, illustrating how the platform provided transparency and recognition of patients’ contributions to a real-world biobank.

## Methods

### Decentralized Biobanking System Design: NFT Digital Twin Framework

**Figure 1:**
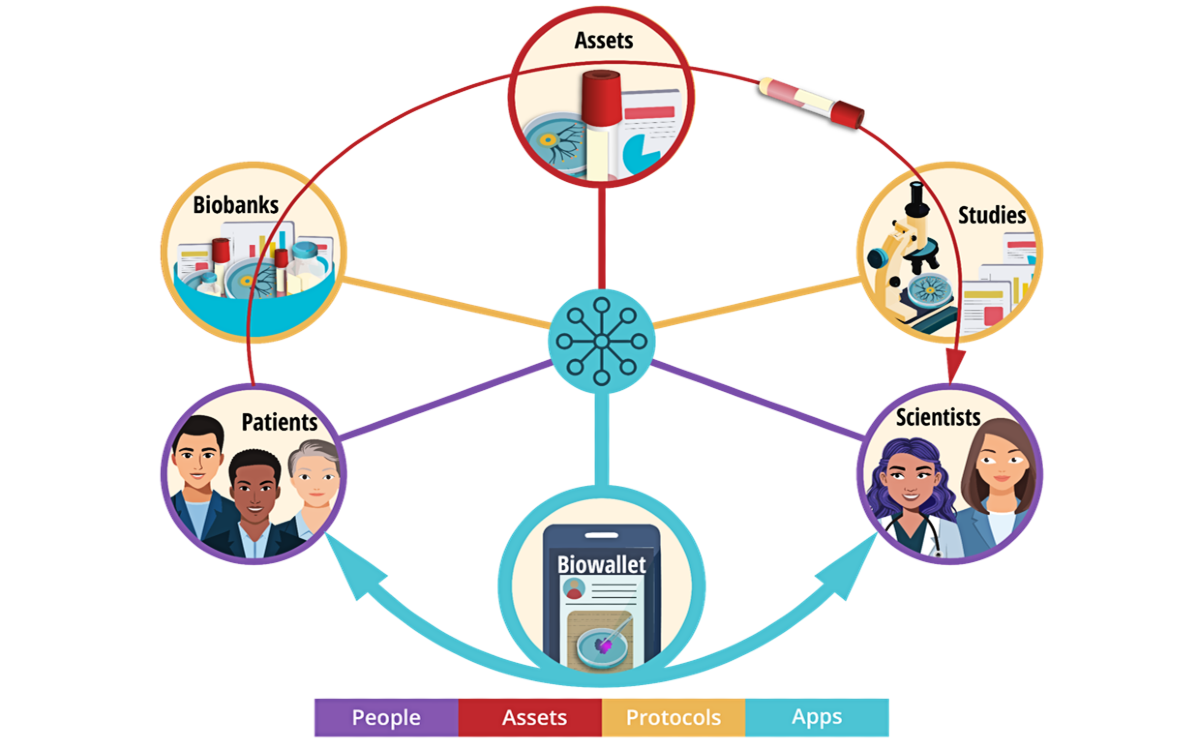
Decentralized biobanking system design—nonfungible token (NFT) framework and software applications uniting patients, specimens, and scientists. This system diagram illustrates key entities of biobanking connected via a specimen supply chain (red arrow) yet presently lacking a unified platform for collaboration. The proposed decentralized biobanking NFT digital twin framework is designed to integrate with this established infrastructure, mapping the stakeholders, specimens, and studies in the biobanking ecosystem and enabling applications whereby they may be united for mutually beneficial collaboration, data exchange, and value-building activities.

Decentralized biobanking builds digital bridges among patients, specimens, and scientists, connecting stakeholders based on real-world relationships predicated upon transactions within existing biobank infrastructure and research protocols (Figure 1). The system design represents all people, protocols, and assets in an NFT digital twin framework, creating a blockchain-backed overlay network on top of the established biospecimen ecosystem. Our approach presents a unique strategy for the progressive inclusion of patients, allowing for the implementation of a composable software platform with programmable, modular elements, mechanisms, and workflows that may be integrated with institutional biobank databases to provide durable transparency without requiring substantial time, labor, or ongoing participation of physicians, biobankers, and scientists. This framework applies privacy by design throughout the engineering process, implementing techniques such as data minimization and innovative system architectures to ensure compliance with established biospecimen collection and research protocols, institutional policies, and data structures. The core benefits of our approach are use case agnostic and can be applied for all biobanks, research protocols, and institutions with minor modifications at each new site.

### Pilot Setting

The Breast Disease Research Repository (BDRR; STUDY19060196) is a large breast cancer biobank platform at the intersection of the University of Pittsburgh, the University of Pittsburgh Medical Center, and Hillman Cancer Center that served as the pilot study use case. Broad prospective consent for the BDRR is embedded in the breast cancer service line, for example, concurrently with surgical consent. Once consented, “leftovers” from any clinical procedures may be collected by the biobank without further notice or engagement. From 2006 to 2023, more than 10,000 patients consented for the BDRR and specimens were collected from 4000 participants to date. In total, approximately 61,000 specimens were collected, and 6000 were distributed for research, with a mix of fresh and frozen distributions. The biobank operates via a hub-and-spoke model, allocating specimens chiefly to local investigators under designated research or subbiobanking protocols (eg, a flagship patient-derived organoid biobank that grows and distributes copies of living 3D cell cultures [approximately n=300]).

### Requirement Gathering

Foundational surveys, semistructured interviews, community engagement, and stakeholder alignment activities with populations with breast cancer, physicians, advocates, and scientists informed our approach to designing a biobanking app for patients [23]. Broadly, we found that patients have an unmet demand for feedback about research on their specimens, with particular interest surrounding personal meaning or potential health benefits for the individual or their family members. For example, a survey respondent noted the following:

Giving patients access to this type of information could decrease the lethal lag between research findings and actual clinical practice.

One patient advocacy leader captured this sentiment, noting the following:

We have been screaming for this, banging on pots and pans. Thank you for taking this on.

Importantly, she alluded to the multifactorial challenge of enabling patients to track and learn about donated biospecimens [23], which would require novel, user-friendly interface designs as well as system architectures and pilot protocols compliant with regulatory norms, compatible with established workflows, and acceptable within the institutional milieu.

Thus, we interacted extensively with the breast cancer service line, the institutional biobanking platform, and institutional review board (IRB) and Office of Human Research Protections leadership, as well as research scientists, clinical and teaching faculty, IT staff, technology transfer teams, and cross-disciplinary institutional leadership. Concurrently, the ethnography of the specimen procurement supply chain allowed us to map the breast cancer biobank ecosystem [23]. We examined all contexts along the data pipeline, from population-level breast cancer screening to diagnostic biopsies and surgical treatments, clinical pathology, and specimen accessioning through the biobanking platform, where it may be stored for future use in –80 °C freezers or distributed fresh for next-generation biobanking applications such as patient-derived organoids, multi-omics, and high-throughput testing. Given the well-documented challenges for biobank sustainability, we took special interest in learning about economic and logistical challenges pertaining to this sector. Regulatory considerations, operational feasibility, and economic analyses will be reported elsewhere [23].

### Prototyping

The first decentralized biobanking prototype established the proof of concept, leveraging ERC-721 NFTs to keep patients connected to donated specimens throughout the research life cycle. The NFT platform was integrated with a novel mobile app for privacy-preserving collaboration among patients, scientists, and physicians in a model breast cancer organoid ecosystem. A second prototype advanced a comprehensive NFT digital twin framework with ERC-1155 modeled using a publicly available real-world organoid biobank dataset (National Cancer Institute Human Cancer Models Initiative) [24,25]. This web-based prototype focused on generating value for scientists, illustrating potential to enhance efficiency, effectiveness, and impact of biospecimen research. Third, no-code front-end mobile app prototypes were developed to demonstrate, test, and refine user interfaces and experiences for the engagement of donors in biobanking.

### User Interface and User Experience

We drafted wireframes using anonymous model biospecimen information from the institutional biobank database. App design processes sought to minimize cognitive effort for mobile app users, maximize accessibility across ages and educational levels, and adhere to rigorous privacy standards and customs in accordance with the established biospecimen collection protocols. We progressively simplified and iterated display text and content to make it as concise and concrete as possible and unified across decentralized biobanking app interfaces. To facilitate navigation, we streamlined presentation of content in each of the 4 core interfaces using accordion elements complemented with individual cards for each biospecimen, with pop-ups to guide transitions within and across interfaces. Unified color schemes, fonts, and item designs adhered to predetermined themes with a standardized format that was gradually refined.

The designs were tested and validated via further research surveys and interviews. Immersive design workshops solidified core app requirements. Initial usability testing included online and in-person sessions with clickable prototypes and functional prototype demonstrations followed by usability testing and cognitive walk-throughs on users’ personal devices.

### Front-End Development and Testing

Finalized mobile app designs were developed using Flutter so that iOS and Android users could participate in the pilot. The apps were tested and deployed to Apple TestFlight and the Google Play Store, allowing for download directly to participants’ personal devices. From August 2022 to January 2023, feedback from 110 unique individuals was incorporated, including 45 (40.9%) BDRR members, 28 (25.5%) who downloaded and tested the app on their personal devices, and 14 (12.7%) who viewed personalized biospecimen content within the app interface. The result was a validated app facilitating interaction between donors and biospecimens within the breast cancer biobank, personalized collection content, and mappings from biobank database details.

### Blockchain Development

Initial decentralized biobanking prototypes were developed experimenting with different tokenization strategies using Ethereum’s ERC-721 and ERC-1155 NFT standards for mapping dynamic relationships among patients, biospecimens, physicians, scientists, and corresponding biobanking and research protocols. However, variable costs of transaction fees (known as gas fees) on the Ethereum network and high friction for blockchain onboarding were major limitations for implementing a real-world pilot.

These constraints informed the design of a functional, blockchain-backed prototype suitable for the pilot population and setting, leveraging a fit-for-purpose blend of centralized and decentralized applications that would enable patients to track and learn about donated specimens appropriate to the highest-order objectives for the first live pilot of decentralized biobanking technology.

A nontransferable ERC-721 NFT, also referred to as a “soul-bound token” [26], was developed to represent each donor’s immutable, inherently unique connection to their personal biospecimens. This token [26] was held within a single *externally owned account* that served as a custodial wallet. Of note, our previous decentralized biobanking prototype for organoid research networks, as described elsewhere, used ERC-1155 to advance a comprehensive digital twin ecosystem with NFTs representing patients, specimens, multigenerational derivatives (eg, patient-derived organoids), scientists, and physicians, as well as externally owned biobanker accounts, demonstrating the potential for a sophisticated solution [25]. However, while using the ERC-1155 standard would have offered savings for deploying multiple token collections representative of the entire biobanking ecosystem, applying them to a single soul-bound token collection for this use case would have yielded no additional benefits while adding unnecessary complexity [25].

Each biowallet NFT served as a customized yet anonymous “token of appreciation” for specimen donation coupled with a front-end user experience simulating token-gated access to personal biobank data. This token-gated process was performed manually, minting the tokens individually via the smart contract interface on Etherscan. Subsequently, the token metadata and transaction details were stored within a secure, IRB-approved database for the eligible user. This created a digital honest broker mechanism for managing in-app participant-specimen engagement without requiring further humans in the loop or revealing donor names or other personally identifiable information to third parties.

### System Architecture

**Figure 2:**
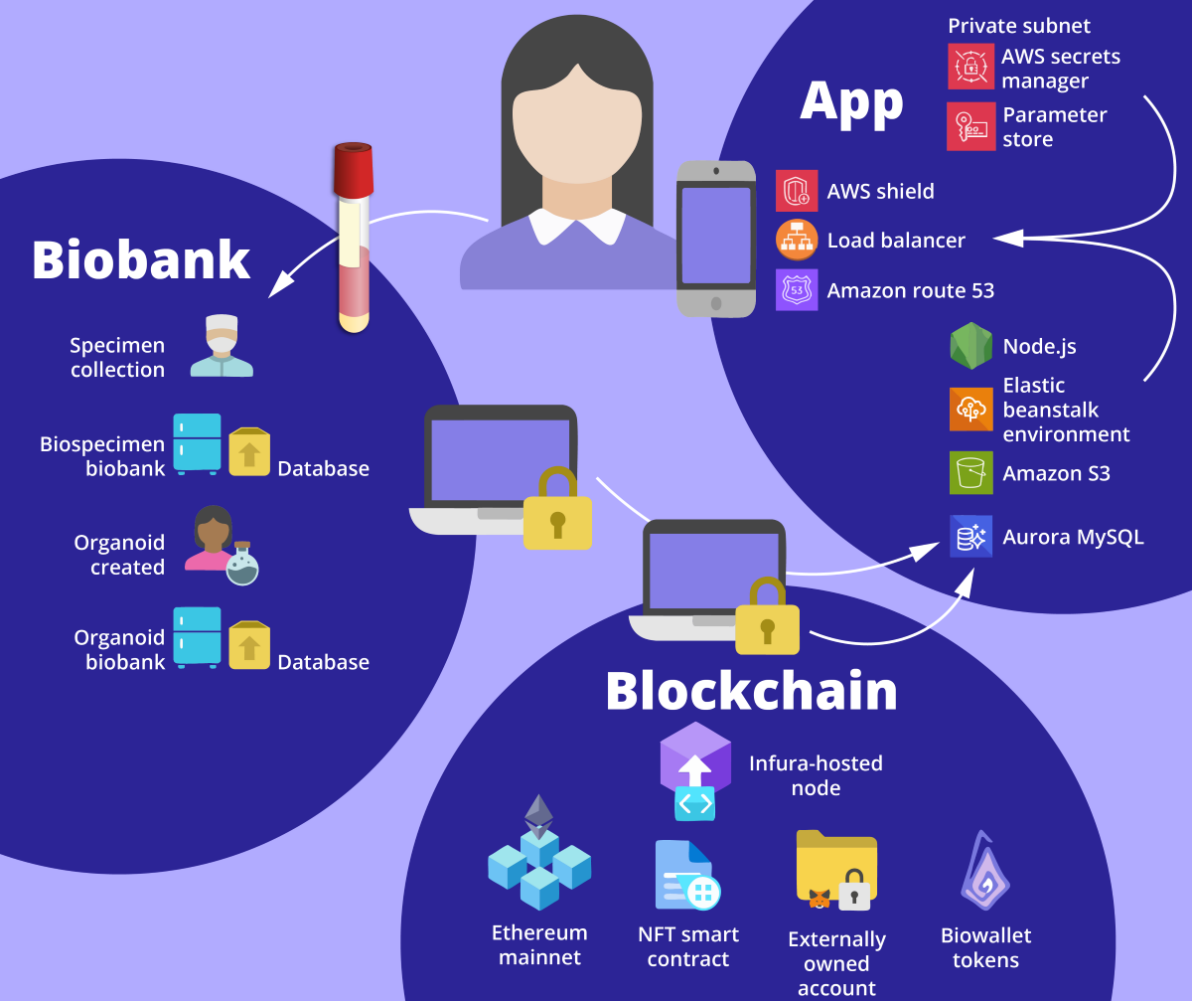
System architecture diagram—decentralized biobanking pilot app for breast cancer biobank. This system architecture diagram incorporates the decentralized biobanking mobile app powered by internal components that handle business logic, data storage, and data integrations built on a cloud-based infrastructure using Amazon Web Services (AWS); this is flanked by corresponding elements connected via secure authorized access devices for interacting with the nonfungible token (NFT) digital framework’s biowallet tokens deployed on Ethereum and institutional data sources from the Breast Disease Research Repository and Institute of Precision Medicine organoid biobank.

The decentralized biobanking pilot system incorporated 3 core components: an *app* overlying *institutional biobank and research infrastructure* with a blockchain-backed *NFT digital twin framework* (Figure 2).

The app used an *n*-tier architecture pattern with interconnected workflows across distinct, modular components with varying responsibilities (Table 1). Our user-friendly mobile app, available on Android and iOS, was powered by applications built using Amazon Web Services. During this initial pilot phase, our system relied on external services and data sources that were not yet directly integrated with our deployed technology. Our NFT framework consisted of an ERC-721 smart contract designed to mint nontransferable, soul-bound biowallet tokens that were deployed to the Ethereum mainnet. Deidentified biospecimen data were provided by biobank personnel to authorized study team members, who would use a secure device to import the records into the pilot system’s database. Both required manual processes for pilot implementation.

**Table 1:** Key details of the decentralized biobanking pilot system architecture.

Component	Technical details
App	Presentation tier: the Flutter mobile app built and deployed using Android Studio (Google) and Xcode (Apple Inc) to enable download to Android and iOS devices. The app provided front-end user interfaces for patients, enabling dynamic interactions, user inputs, and the presentation of queried information from institutional data sources through the app tier. Google’s Firebase Authentication services manage account creation and management, encrypting data in transit using HTTPS and at rest using the scrypt standard cryptographic protocol. Passwords are stored securely using encryption, salting, and 1-way hashing following NIST^a^ 800-63b recommendations. App tier: used a Node.js (OpenJS Foundation) server to enable all core functionality and logic of the app, including specimen tracking with enhanced transparency into biobank activities and subsequent research. This layer is also responsible for enforcing security and access rules, handling connectivity to and communication with data sources and external services, and processing data to return to the presentation layer. Deployed on AWS^b^ Elastic Beanstalk, the app instances sit behind load balancers for scalability, running in private subnets. Data tier: hosted by an Amazon Aurora database cluster using the MySQL engine. It hosts a secure, highly available database that stores and retrieves the information necessary for the app to run. This includes donated sample records housed on the BIOS^c^ and corresponding biospecimen freezer repositories across 4 physical locations of the Pitt Biospecimen Core, as well as unique cryptographic IDs from Firebase and claimed biowallet NFTs^d^ to establish privacy-preserving data linkages between donors and their deidentified biospecimens. As noted in the presentation tier, user credentials for accessing the app are stored separately on secure Firebase servers. Infrastructure tier: referenced within the app and data tiers, our AWS cloud infrastructure provides the foundation for networking and security, ensuring availability, scalability, and interoperability across system components Multimedia Appendix 1.
Blockchain	NFT framework: an ERC-721 smart contract designed to mint nontransferable, soul-bound biowallet tokens was deployed to the Ethereum mainnet via a transaction sent to an Infura-hosted node from a local Node.js runtime environment using Hardhat. The overarching framework incorporates NFTs representing all stakeholders, specimens, and protocols, allowing for composable layers of complexity, utility, and value to be built upon the PIO^e^ architecture.
Biobank	Institutional biospecimen and research databases: biobank personnel provided access to deidentified biospecimen data via OneDrive Microsoft Excel (Microsoft Corp) files to an authorized study team member, who would use a secure device to import the updated records into the Aurora database. Similarly, Microsoft Excel files containing biobank (BDRR^f^) registered members were provided by research staff as exported from OnCore. In addition, imaging and research data from an organoid biobank “spoke” were shared via OneDrive, and curated representative datasets were hosted on Dropbox (Dropbox, Inc).

^a^NIST: National Institute of Standards and Technology.

^b^AWS: Amazon Web Services.

^c^BIOS: Biospecimen Inventory and Operations System.

^d^NFT: nonfungible token.

^e^PIO: programmed input-output.

^f^BDRR: Breast Disease Research Repository.

### Pilot Study

Participants were recruited via electronic and paper fliers for “Decentralized Biobanking “de-bi”: An App for Patient Feedback from Biobank Research Donation” (STUDY22020035). The pilot aimed to recruit 300 participants over 6 to 12 months. App download invites were distributed via email with Apple and Android instructions. IT support was provided as needed, with real-time bug fixes and improvements based on user feedback. App interfaces, design, and features were iterated in monthly sprints. Participatory research, user-centered design, and usability testing, as well as quantitative and qualitative assessments of patient, physician, and scientist acceptability, will be reported elsewhere. NFT minting for pilot performance took place from March 7, 2023, to May 8, 2023. Multimedia Appendix 2 details the pilot recruitment to sample tracking process.

### Data Sources and Analysis

The technical data reviewed included conceptual models, technical diagrams, product feature documentation, and screenshots of user journeys as experienced by decentralized biobanking pilot participants using the Flutter app. We also consider biospecimen collection data from the institutional Biospecimen Inventory and Operations System via Microsoft Excel (Microsoft Corp) exports, in-app activity data recorded in a MySQL database, and blockchain transactions on the Ethereum network accessed via Etherscan. Technical feasibility was assessed from feature requirements, interface designs, and quantifiable user experiences from the live implementation. To further evaluate pilot outcomes, we provide simple descriptive statistics from the quantitative datasets and comparative cost analyses for alternative NFT design strategies calculated using values from tokens minted during the pilot. Patient experiences were captured via written feedback from a co-design workshop during the app development phase and a usability workshop session held with pilot participants.

### Ethical Considerations

Research was performed under IRB-approved human subjects research protocols and a Quality Improvement protocol (Textbox 1 provides protocol numbers, titles, and approving body). Participants provided informed consent or the equivalent, in accordance with respective protocols. Conflict of interest disclosures were included in consent documents and verbal disclosures were provided for all online and in-person encounters. All data reported here are either de-identified or anonymized and privacy-by-design was utilized within the de-bi app to maintain confidentiality of participant identities. Participants were not compensated for participation in the biobank, stakeholder interviews, quality improvement activities or de-bi app pilot study (STUDY19060196, IRB00019273, QRC 3958 and STUDY22020035, respectively). Our foundational research protocol (STUDY22010118) provided $10 gift cards for surveys, with an additional $20 for those who completed follow-up interviews.

**Human participants and quality improvement protocols for technology feasibility.**

- STUDY22010118: patient views, preferences and engagement in next-generation biobank research (University of Pittsburgh)
- IRB00019273: nonfungible tokens for ethical, efficient and effective use of biosamples (Johns Hopkins University)
- STUDY19060196: Breast Disease Research Repository: tissue and bodily fluid and medical information acquisition protocol (04-162; Hillman Cancer Center)
- QRC 3958: patient-facing biobank platform development Quality Improvement proposal for Beckwith award–breast cancer supply chain analysis, biobank token model development, and initial pre-pilot testing with University of Pittsburgh Medical Center patients (University of Pittsburgh Medical Center)
- STUDY22020035: decentralized biobanking “de-bi”: exploring patients interests in feedback, education, follow-up, engagement and tokens of appreciation regarding biobank donation via mobile and web applications (University of Pittsburgh)

## Results

### Prepilot Results

A co-design session (n=15) was conducted before the pilot to characterize patient preferences and areas of confusion. This session was one in a series of extensive participatory design sessions, which we have reported elsewhere [23]. Participants were most excited about decentralized biobanking for feedback and recognition (“to see my own cells+know how those cells are advancing science”), community-engaged research (“to connect with others through this app”), and precision medicine potential (“to get helpful results regarding my health”), suggesting acceptance of our vision and overall approach. At the conclusion of this phase, there was still confusion surrounding logistics and governance (“how we *find* our samples and approve their use”), technical concepts (“Why NFT’s?”), and unanswered big-picture questions (“Short+long-term—who benefits from this?”) regarding the decentralized biobanking platform. Table 2 provides a thematic overview and representative quotes.

**Table 2:** A thematic overview of participant feedback gathered through a prepilot co-design session.

Theme			Prepilot participant feedback
**Aspects participants were “most excited about”**			
	Personalized feedback and recognition	“The opportunity to see my own cells+know how those cells are advancing science and clinical care.” “Having knowledge about [sample] types, research and current news about my tumors.” “To be able to follow where my personal donation goes, and what they are doing with it, and what they get out of it.”	
	Community-engaged research	“Great for mutation studies with multiple primary cancer+tumors.” “Keeping up to date with genetic mutation research.” “I’m excited to connect with others through this app.” “That patients who invest their tissue in research are able to connect as co-investigators.”	
	Potential health benefits	“I’m excited about the idea that there may be more ways to care for my family—better research practices may enable the medical field to work smarter—maybe ensuring that my children don’t need surgery, chemo, etc.” “I am very excited for anything that can improve my health and outcome (and of others).” “Being able to get helpful results regarding my health.” “I’m excited about the possibility to know how my tissue reacted to a treatment.” “Patient access to personal info/data; Personalized medicine potential.”	
**Aspects participants “still found confusing”**			
	Big picture	“Why do people still get cancer, dammit!” “I don’t understand 1) How this may really help me+my family, 2) Short+long-term—who benefits from this? 3) Where does the $ come from? 4) What are we giving up/sacrificing by saying ‘yes.’” “How will Dr. utilize?”	
	Logistics and governance	“I don’t understand how we find our samples and approve their use—I also don’t understand what studies we could ‘suggest’ or enable through the samples we have provided.” “How likely is it that my samples will be used?” “Can you use it [de-bi app] even if your surgery already happened?” “How to get my tissue submitted to researchers.”	
	Unclear technical terms and concepts	“Not really sure what an organoid is—is it a picture/video of my actual cells or is it a model of my cells?” “Why NFT’s?” “I am still learning about NFTs and how they will help breast cancer patients.” “How will patients interpret data—will it be translated?”	

### Overall Pilot Results

#### Overview

**Figure 3:**
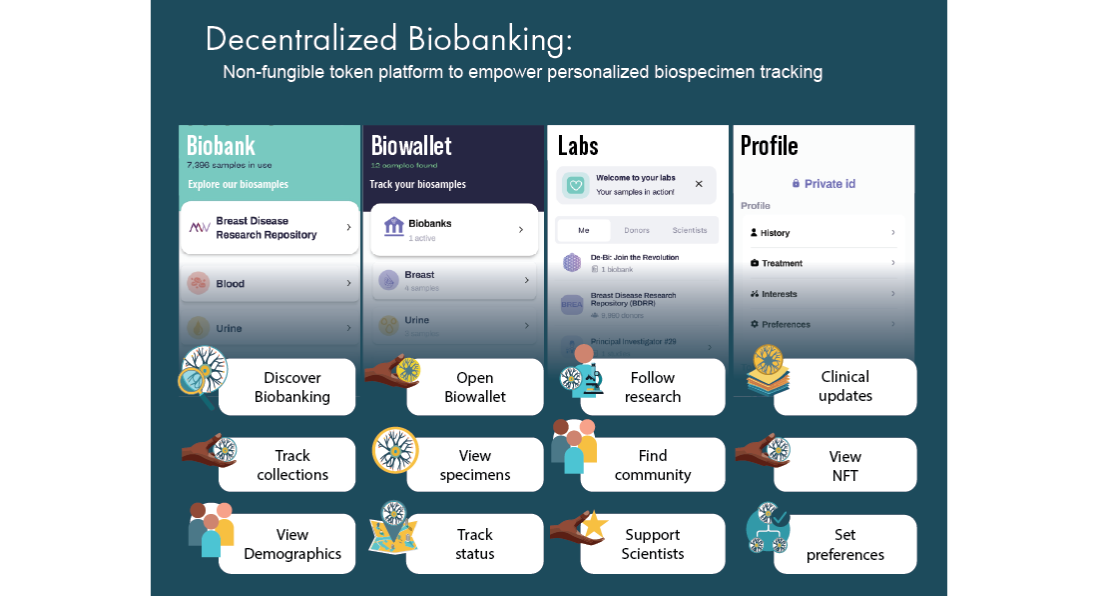
Decentralized biobanking platform user journey. The user journey map demonstrates the status quo of the patient experience with biobank donation as well as the 4 key features of the decentralized biobanking mobile app that was piloted for a large breast cancer biobank member population from January 2023 to May 2023. Each of the columns represents primary activities within the different core screens of the decentralized biobanking mobile app, which the invited participants downloaded to personal iOS and Android devices. The Biobank, Biowallet, and Profile sections are illustrated with key activities and features. The Lab section on the far right is illustrated, although the journey for the community engagement feature is outside the scope of this study and is addressed elsewhere. NFT: nonfungible token.

Over 10 weeks of active recruitment (February 16 to April 30, 2023), 1080 unique participants enrolled in the decentralized biobanking pilot, including 9.54% (930/9750) of confirmed biobank members (Multimedia Appendix 3). Approximately 600 app invites were distributed, and 405 participants downloaded and completed app registration, including 361 (89.1%) biobank members. All app users were female (405/405, 100%), and the mean age was 56 (SD 12.8; range 18-87) years, making them younger than both the broader biobank membership and decentralized biobanking pilot participants (mean ages of 64, SD 13.6 and 58, SD 13.1 years, respectively). Multimedia Appendices 4 and 5 detail pilot participant and app user characteristics relative to those of the overall biobank membership. There were 4 key features of the piloted app, as shown in the user journey map (Figure 3). Biobank, biowallet, and profile features and quantified user journeys are illustrated in subsequent Journey sections, and laboratory features and respective user journeys for that context are also described in detail elsewhere.

#### Journey 1: App Onboarding and Biowallet NFT Minting Process

**Figure 4:**
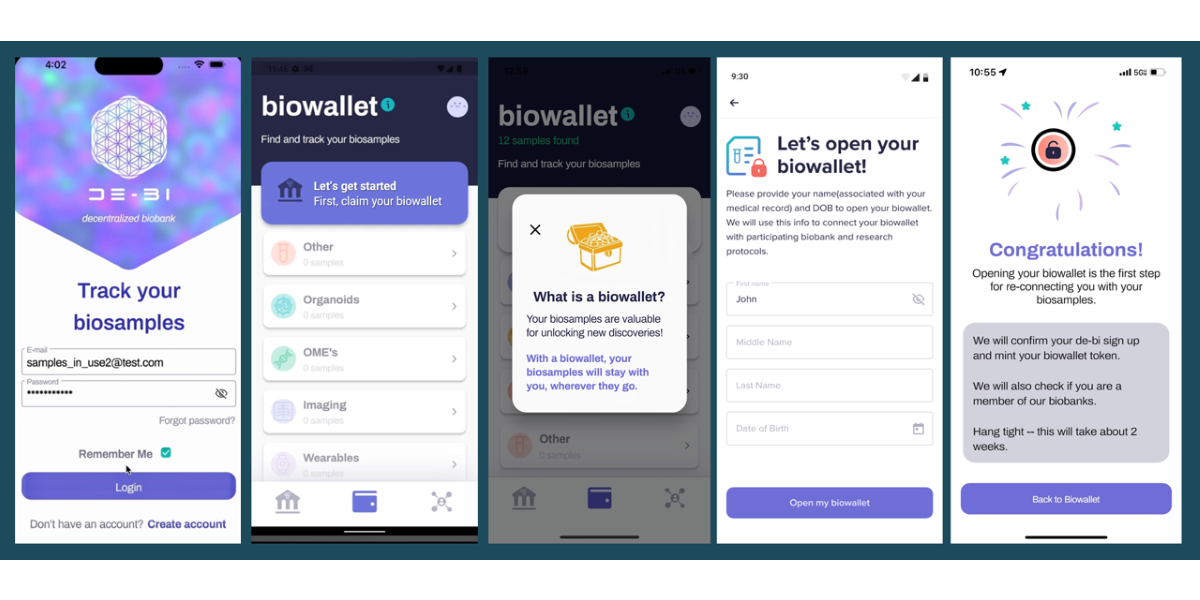
Opening a biowallet—simulation of token-gated specimen access. The process of opening a biowallet required participants to enter their name and date of birth, triggering the system to match participants to corresponding members in the biobank (Breast Disease Research Repository). Once specimen status was established, biowallet nonfungible tokens were minted, specimens were linked to the account, and email notifications indicated to participants that their biowallet was available.

Upon downloading the app, users entered their name and birth date, triggering verification of biobank membership and sample collections, with “biowallet NFT” minting, if applicable, serving as a digital representation of membership in the biobank donor community, delivering a user experience of a token-gated bridge between the user’s app and specimen data, if available (Figure 4).

##### Simulated Token Gating Workflows

Once users entered their name and date of birth into the decentralized biobanking app, a manual, coordinated effort involving biobank personnel and authorized study team members verified each user’s biobank consent and matched donors to their respective biospecimens via a unique anonymous study ID linked to a Firebase (Google) unique ID associated with their decentralized biobanking app account. During this process, study team members would also mint a unique biowallet token for each verified donor with specimens. These tokens were held in a custodial wallet, but each token identifier was linked to donor records within the Amazon Aurora database to establish a second privacy-preserving mechanism for data linkage.

Firebase established the functional linkage to allow for proper access control and permission management within the app for this pilot, whereas the biowallet NFTs and the act of claiming were representative as a proof of concept as well as a token of appreciation for participating donors. This decision was made to limit excess complexity related to using web3 technologies as a barrier to participation for this population while providing a comprehensible introduction to the concept of NFTs for establishing relationships between donors and their samples. Our aim was to ensure that donors were not excluded from engaging with the platform based on the extent of their blockchain expertise.

**Figure 5:**
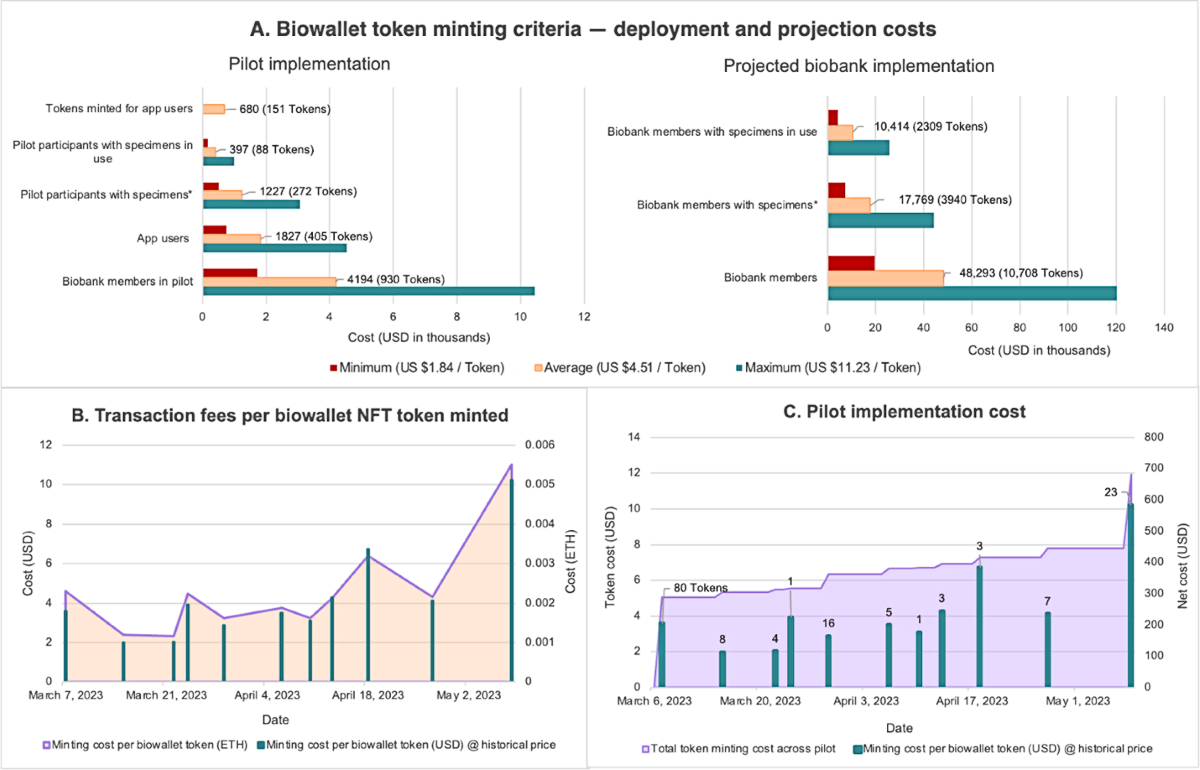
Nonfungible token (NFT) minting costs and calculations for the breast cancer biobank pilot. (A) Pilot implementation—comparison of biowallet token minting criteria for the total cost of pilot deployment. Cost analysis used variation in token minting costs observed throughout the pilot study to model minimum, average, and maximum costs (US $1.84, US $4.51, and US $11.23, respectively). *Selected token minting criteria for the decentralized biobanking pilot. (B) Transaction costs in US $ and ether (ETH) are illustrated for 151 NFTs minted during the decentralized biobanking pilot. (C) Timeline mapping variable cost of biowallet minting events and cumulative costs of minting 151 NFT biowallet tokens throughout the decentralized biobanking pilot.

Various criteria for minting Biowallet tokens were considered for entire pilot and biobank deployment. Using variation in token minting costs observed throughout the pilot study to model minimum, average, and maximum costs (US $1.84, US $4.51, and US $11.23, respectively), the selected model, minting tokens for all 272 pilot participants coenrolled in the biobank with one or more specimens collected, was projected to cost US $1226.72 (SD US $41.91; range US $500.48-$3054.56, Figure 5A, left). Extended entire biobank implementation, this model is projected to cost US $17,769.40 (SD US $159.52; range US $7265.62-$44,229.27; Figure 5A, right). Other models, such as specimen distribution to a research protocol or biobank membership were also considered.

##### Token Minting Costs

The cost of deployment of the biowallet NFT protocol on Ethereum was US $223.52. A total of 151 biowallet tokens were minted for US $680.49 at an average of US $4.51 per token (SD US $2.54; range US $1.84-$11.23; Figure 5B. Biowallet tokens could be requested by decentralized biobanking pilot participants who downloaded the app and had one or more specimens collected (148/405, 36.5%). For context, procurement, processing, storing, and disbursement of biospecimens in this institutional biobanking platform costs an estimated US $1600 per case.

Biowallet tokens could be requested by decentralized biobanking pilot participants who downloaded the app and had one or more specimens collected (148/405, 36.5%). During the pilot, 140 total tokens were requested and minted for eligible participants. Minting events varied in cost based on fluctuating transaction fees and the number of participants who had requested biowallet tokens since the last token minting event. For instance, minting events ranged from US $3.11 for minting one token, to US $288.52 for minting 80 tokens in the first batch (Figure 5C).

#### Journey 2: Biobank Orientation and Research Profile

**Figure 6:**
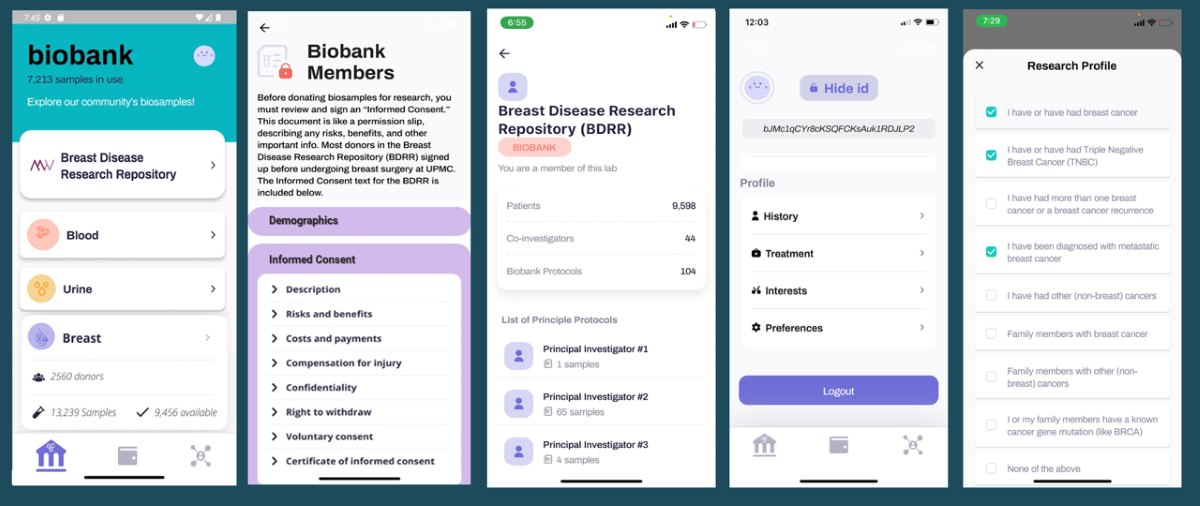
Biobank orientation journey, illustrating the biobank screen and user workflow introducing app users to biobank processes, what it means to be a biobank member, and regularly updated snapshots of investigator activities, protocols, and specimen allocations, at the level of the overall bank. The biobank also linked to participant's personal research profile, where they could provide key clinical details, interests, and preferences related to research on their specimens.

After requesting a *biowallet***,** users were directed to visit the *biobank*, where they were oriented and learned about the overall biobank inventory and activities, including demographics of the consented donor population, framed as “biobank members”; informed consent content; principal investigators; and respective biobank operations and research activities for entire specimen collection (Figure 6). We included education about research protocol development, IRB oversight, procedures for specimen allocation, and investigator- and protocol-level transactions. The biobank displayed 60,973 biospecimens from 3940 unique donors collected from February 1995 to May 2023 and updated on a regular basis, with 318 new specimens added during the pilot. The feature tracked collection and distribution totals for the biobank, with breakdowns for each specimen type (Table 3).

The “profile” allowed participants to enter clinical history and treatments relevant for research on their specimens. We also assessed research interests, privacy preferences, engagement interest, and willingness to donate additional specimens to scientists as needed. In total, 37.8% (153/405) of the app users completed one or more portions of the profile, including 37.1% (134/361) of the biobank members. The profile also displayed the random “Private ID” number, which enabled users to remain deidentified while linking to their respective specimens. During the pilot, we experimented with the naming conventions, location, and order of presentation of biobank and profile features to assess impact on participants’ understanding of the biobank environment, affordances, constraints, and opportunities presented by the decentralized biobanking platform.

Nearly all participants who filled out the research profile (151/153, 98.7%) added one or more clinical details (eg, familial history of breast cancer; Multimedia Appendix 6). Profiles were completed by 39.9% (59/148) of the participants with samples, collectively annotating 886 specimens, including 760 (85.8%) available for future use, 36 (4.1%) “on hold” for a designated protocol, and 90 (10.2%) that were distributed for research, with information that was not contained within the institutional biobank database. In addition, participants added preferences regarding specimen use, willingness to provide further data and specimen donations, and future research engagement.

**Table 3:** Decentralized biobanking pilot population, app user and token claiming overview.

Pilot population		App users, n (%)^a^	Token claimed, n (%)^b^
**Total (N=1080)**		405 (37.5)	130 (12.04)
Biobank members (n=930)^c^		361 (38.82)	128 (13.76)
Biobank members with specimens (n=272)^d^		148 (54.41)	125 (46)
Collected specimens (n=3904)^d^		2133 (54.64)	1812 (46.41)
**Biobank members with specimens in use (n=165)^d,e^**		88 (53.33)	74 (44.85)
	Fresh (n=90)	46 (51.11)	40 (44.44)
	Frozen (n=100)	50 (50)	40 (40)
**Specimens in use (n=377)^d,e^**		202 (53.58)	177 (46.95)
	Fresh (n=195)	104 (53.33)	95 (48.72)
	Frozen (n=182)	98 (53.85)	82 (45.05)
**Number of donors with specimens available (n=242)^d^**		132 (54.55)	110 (45.45)
	Breast (n=147)	81 (55.1)	67 (45.58)
	Blood (n=185)	97 (52.43)	82 (44.32)
	Urine (n=166)	91 (54.82)	80 (48.19)
**Specimens available (n=3309)^d^**		1757 (53.1)	1522 (46)
	Breast (n=345)	205 (59.42)	178 (51.59)
	Blood (n=2172)	1145 (52.72)	988 (45.55)
	Urine (n=783)	406 (51.85)	355 (45.34)

^a^Specimen values and donor counts for all app engaged participants with specimens collected.

^b^Specimen values and donor counts for all app engaged participants with specimens collected who claimed biowallet tokens during the pilot study.

^c^Donor counts for all biobank consented pilot participants.

^d^Specimen values and donor counts for all biobank consented pilot participants with one or more specimens collected.

^e^Specimens considered in use if distributed to a research protocol as of May 4, 2023. A total of 218 specimens among all pilot participants with collected specimens designated “on hold” for future research use are not shown.

#### Journey 3: Claiming and Viewing the Biowallet NFT

##### Overview

Linking app accounts to biospecimen data occurred offline and took up to 2 weeks supported by software scripts and manual processes, including checks for false mismatches (eg, due to typos). Once biowallet NFTs were available, email notifications prompted participants to log in to their decentralized biobanking app to open their biowallet and access their personalized biospecimen data.

**Figure 7:**
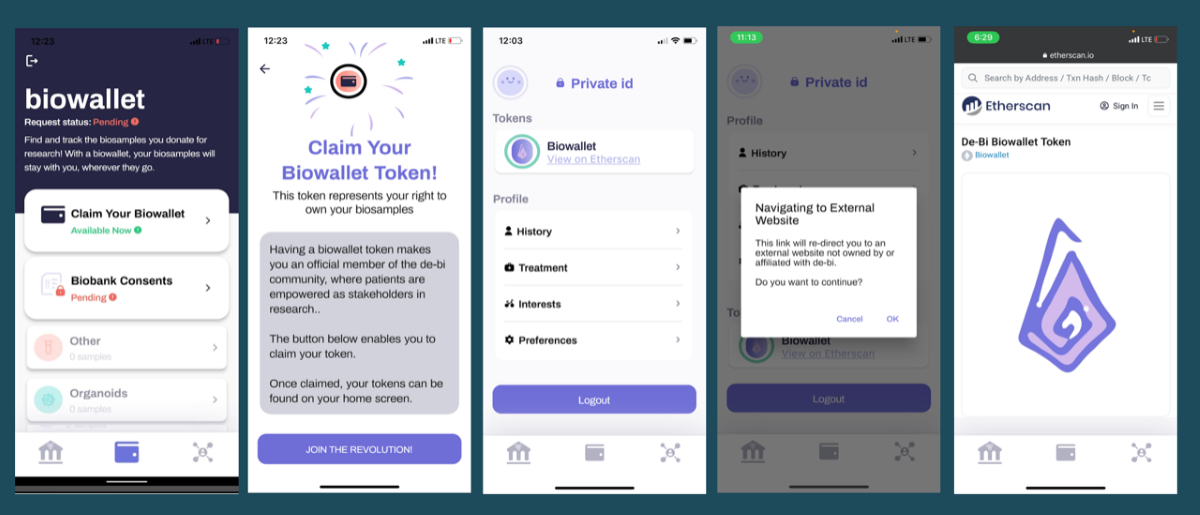
Claiming and viewing the biowallet nonfungible token (NFT). The figure illustrates the biowallet NFT claiming process, first showing the appearance of the biowallet when the token is available to be claimed. Next, the claiming process is shown, which invites donors to “join the revolution!” Once claimed, the user’s personal NFT is represented on the profile page, which is connected via a hyperlink and an in-app display of the Etherscan view of the NFT, a customized biowallet logo for the pilot, and corresponding blockchain transaction data.

Once claimed, the “Biowallet token” appeared on the bottom of the screen with a link to view the corresponding Ethereum transaction data (Figure 7). The profile screen showed how patients could add clinical details that are not in the biobank database, making their biospecimens more readily discoverable by prospective users, reducing reliance on third-party chart review during study planning. The biowallet NFT signified membership in a collective committed to breast cancer research. Once claimed, the individual’s unique biowallet NFT could be viewed via an in-app Etherscan display. The app user experience represented this process as a symbolic “token of appreciation” as a form of reciprocity for biobank contributions.

##### Proof of Concept for Blockchain-Backed Biobanking App

The initial round of minting included “tokens of appreciation” for participants who were active in the demonstration phase of the app design and usability testing. The blockchain mechanism was initially tested with 4 test mints followed by minting “tokens of appreciation” for 7 demonstration phase participants. In total, 71% (5/7) of the demonstration users successfully completed the token minting claiming process, illustrating the use of the “biowallet” NFT as a representation of the individual’s membership in the biobank community. After validating functional integration of the blockchain simulation, eligibility for biowallet tokens was limited to those with confirmed specimens in the breast cancer biobank, enabling us to simulate use of the NFTs to establish token-gated access to deidentified specimen accounts.

**Figure 8:**
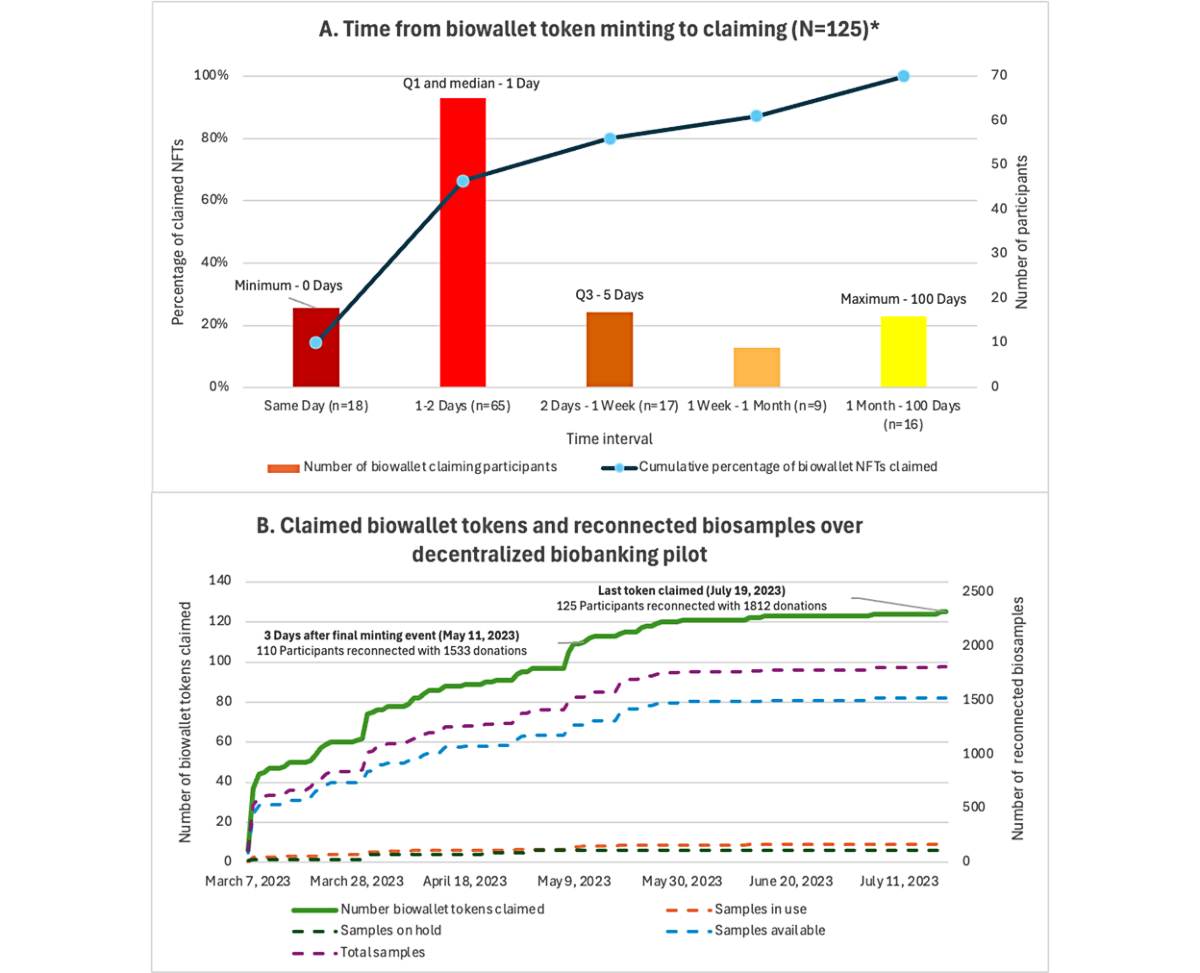
Nonfungible token claiming details for the decentralized biobanking breast cancer biobank pilot. Participant engagement and timing illustrates (A) interest in biospecimen tracking and receptiveness to email notification to facilitate the token claiming process and (B) the effective reconnecting specimens to participants that occurred during the pilot as tokens were claimed. In total, 89.3% (125/140) of tokens minted for app users with specimens were claimed during the pilot. Tokens were considered unclaimed after ~2.5 months following the final token minting event. A total of 15 participants had not yet claimed their token as of the conclusion of the pilot.

Of 148 app users with specimens, 140 (94.6%) initiated the biowallet token minting process during the pilot. Of 140 tokens minted, 125 (89.3%) were claimed by users, with an average of 10 (median 1, IQR 1-5, range 0-100) days between token minting and token claiming (Figure 8). Compared to individuals who did not claim their biowallet, those who did claim their biowallet were slightly younger (average of 58.9, SD 10.8 vs 61.9, SD 14.3 years) and had a similar time since biobank consent (7.8, SD 5.0 years since consent for claimants vs 7.7, SD 5.3 years for nonclaimants; Multimedia Appendix 7).

Ethnography of the US cancer specimen supply chain, including engagement with industry and academic stakeholders, generated the following conservative estimates for the commercial value of cancer tissue, blood, and urine specimens with well-annotated clinical data: US $1000 for cancer tissue, US $500 for blood, and US $300 for urine. Hypothetically, this equates to US $1 million of “available” specimens being populated into app users’ biowallets during the pilot. Similarly, this corresponds with a total value of approximately US $30 million for unused specimens in frozen storage, with roughly US $7000 in value per specimen contributor. Additional details of the scalability and economic feasibility of the proposed blockchain solution will be addressed elsewhere.

#### Journey 4: Viewing Personal Specimen Details

**Figure 9:**
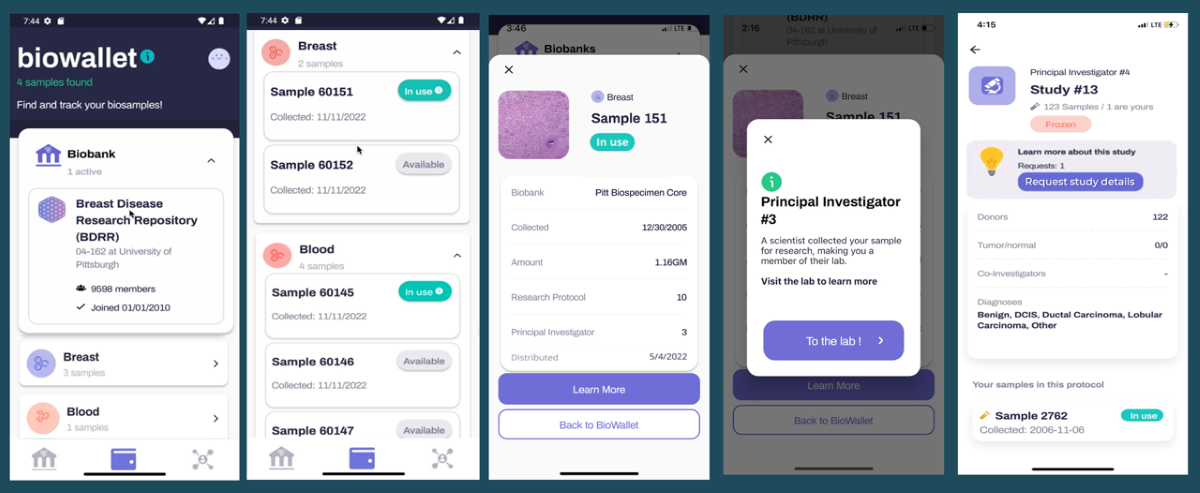
Biowallet sample tracking journey. This figure illustrates the participant experience learning about their personal specimen donations via an interactive biowallet landing page. Pop-up and accordion features enabled participants to learn about their specimens, including the type, collection date, distribution to a research protocol versus availability for future use, and explore further details about similar donations and distributions.

The “biowallet” was where participants could view details about when they consented for biobank donation (Figure 9). Once linkage between the user’s app and respective biobank data was established, individuals were able to track and learn about their own biospecimens. Details available via an interactive accordion feature included their biosample collection date, sample type and medium, if and when each sample was shared for a particular research protocol, and similar sample-level information within the institutional database. The biowallet also includes a taxonomy of physical and digital biospecimen data types that may, in the future, be trackable by individual participants.

Further details regarding specimen distribution and availability were indicated via additional pop-ups, providing users with an opportunity to navigate to an app-based laboratory. Here app users could learn how many donors had contributed specimens of similar types, or had specimens distributed to the same research protocol. Of the biobank members using the app, 41% (148/361) had their “biowallet” populated with a total of 2113 specimens (mean 14.4, SD 12.1; range 1-84), including 1414 (66.9%) blood specimens, 419 (19.8%) urine specimens, and 296 (14%) breast tissues. In total, 70.9% (105/148) of sample holders had one or more breast tissue specimens. A total of 59.5% (88/148) had one or more specimens “in use” (mean 2.3, SD 1.6 per person; range 1-8), 40.5% (60/148) of the participants with specimens had none “in use,” and 4.7% (7/148) of the participants had specimens “on hold” (mean 24.9, SD 16.3; range 10-61). Individuals who had no specimens available received a digital biobank membership card (Figure 9, panel 2) and in-app text notifying the participant that no specimens had been located (yet), with a range of possible explanations.

Collectively, 202 of app users’ specimens were “in use,” including 104 (51.5%) that were delivered “fresh” the day of donation (eg, for organoid development) and 98 (4%) from a frozen collection. A total of 8.2% (174/2113) were “on hold” for a designated study, and 83.15% (1757/2113) were “available.” App users’ specimens were distributed to 22 different investigators under 42 research protocols. Between February 15, 2023, and May 4, 2023, users donated 39 new specimens, which appeared on the app, 2 (5%) of which were distributed fresh. In addition, 18% (7/39) were distributed from frozen storage, and 54% (21/39) were placed “on hold” during the pilot.

#### Participant Feedback During the Pilot

During the pilot, cognitive walk-throughs with participants illuminated areas of interest along with potential opportunities for design improvement. Key areas of excitement included seeing how their samples were used. One participant stated the following:

I will [otherwise] never know anything about my cells.

Areas for improvement included improving technological accessibility (eg, making it iPad compatible) and clarifying the information presented (eg, “Will there be a way to learn more about each study?”). Table 4 provides a detailed thematic overview and representative quotes.

**Table 4:** A thematic overview of participant feedback gathered through cognitive walk-throughs conducted during the pilot.

Theme		Pilot participant feedback
**Things they liked**		
	Big picture	“This is cool on so many levels.” “Incredible concept to learn about.” “There are endless possibilities and uses for this.” “There is hope for others by giving my cells.”
	Personalized feedback	“I can’t wait to see what’s being done with my samples!” “Loved the idea of having access to my tissue info+how the two cancers are connected.” “I will [otherwise] never know anything about my cells.” “I’ll get to see the process.”
	Empowerment	“Information I could never access before.” “Give patients more control and information.” “Profile preferences—great idea.” “Private ID+Ability to connect w/ others in similar diagnosis.”
	User interfaces and user experience	“Menus under biowallet are clear+concise.” “Look of the app.” “Easy to navigate.” “Easy to use/menus good.” “Love the status of ‘in use’ and ‘available.’”
**Things they did not like or that did not meet their expectations**		
	Information provided	“Where are investigators that have my tissue or samples.” “Unclear when no samples (needs explanation).” “Will there be a way to learn more about each study?” “I need a little more background before fooling around with the app.”
	Accessibility	“Needed tutorial.” “Are there options for people who do not have email on their phone.” “Under personal history, other than TNBC (triple negative breast cancer) other breast cancers should be identified.” “Need to be able to use on an iPAD for larger screen.” “Possible to put app on android tablet?” “Being older I’m not a techie and it takes a while.”
	Functionality and user navigation	“Biowallet should be first icon.” “Make biobank/wallet first tab.” “Add search bar in connect.” “Some functions are more intuitive than others—more prompts are needed.” “What was the purpose behind ‘home’ icon community samples.”

## Discussion

### Principal Findings

The decentralized biobanking pilot demonstrated the technical feasibility of design, development, and implementation of a user-friendly app to deliver transparency and engagement for donors to a well-established biospecimen collection protocol at a US academic medical center. Over 400 participants downloaded and tested the decentralized biobanking app during the pilot, asserting interest in tracking their biospecimens, demonstrating the usability of a patient interface for institutional biobanking data. “Biowallet” tokens (ERC-721) were minted for app users with confirmed specimens, and 89.3% (125/140) successfully claimed their NFTs on the app, with over half (72/125, 57.6%) of the population achieving the task within 1 day of token minting.

Pilot participants’ biowallet token claiming process symbolically asserted their right to know what happens to their inherently unique biospecimens, to which they are immutably linked via a nontransferable, one-of-a-kind relationship. The user experience simulated an NFT-gated process, functionally reconnecting app users to >1800 deidentified specimens, providing visibility of affiliated community members and related research activities all while preserving confidentiality. Critically, this was achievable with data architecture, interfaces, and workflows that maintained compliance with preexisting deidentification standards and specimen collection and distribution protocols.

Similarly, we showed how integration with institutional biobank infrastructure can passively provide transparency for donors without imposing undue burdens on investigators or relying on individual research programs to sustain community engagement. Transparency in biobanking has the potential to rebuild donor trust in biobanks and improve accountability in biomedical research [27-29]. Consequently, transparency may be a driver to improve biobank donations, particularly among communities with historically rooted distrust of biomedical research [30,31]. The decentralized biobanking framework also allowed for the retrospective and prospective onboarding of donors, demonstrating the potential to convert existing biobanks to a progressively decentralized, patient-centered model.

Minting biowallet NFTs averaged US $4.51 (SD US $2.54; range US $1.84-$11.23) per token, with a projected total cost of US $17,769.40 (SD US $159.52) for all biobank members with specimens. Importantly, a 1-time minting expense of <US $5 per patient may be considered marginal, especially in view of the cost of specimen procurement, storage, and distribution. A workshop on biospecimen economics found the cost of operating a large biobank to be US $861 per patient [32]. The value of the specimens themselves is also substantial relative to minting expenses; academic researchers may pay up to US $200 per sample, whereas commercial entities may pay up to US $20,000 per sample [32]. When biospecimens are converted into living models (eg, organoids), the expenses of both processing and development increase, but the value is multiplied several-fold as 1-mL aliquots of the model may cost upward of several thousand dollars per copy for academic and commercial users alike [33,34].

Importantly, we also demonstrated how empowering patients may in turn help scientists by allowing them to annotate their biospecimens with relevant data that may not be represented in the institutional biobank database or may be otherwise not directly available to prospective or current specimen users. Over 37% (150/405) of the participants demonstrated how longitudinal donor involvement might be leveraged to improve biosample curation and discoverability, creating opportunities to enrich research; link siloed datasets; and drive more efficient, community-driven use of biobank resources. Enhanced annotation of biospecimens with clinical data reflects increasing demand among the biobanking community to gain more contextual biospecimen data [35]. Project LUNGBANK is an example of ongoing efforts to provide more comprehensive clinical data to enrich biospecimens [36]. In LUNGBANK, clinically relevant findings collected through manual chart review of patient medical records were used to annotate biospecimens [36]. For the decentralized biobanking app, more intuitive, strategic placement of the profile feature and improved framing of its functionality and benefits for donors and scientists will be essential to optimize the utility of this feature.

Although relatively limited in functionality compared to the NFT framework advanced in our preclinical prototypes, the blockchain aspect of the piloted app was significant for several reasons. First, it represents the first time that most of our participants, including several octogenarians, had ever interacted with blockchain technologies. Second, persistence in overcoming the friction of onboarding related to the blockchain elements served as further evidence of the high value that patients place on tracking their specimens, to the point that they were willing to participate in a cumbersome, multistage process that, in some cases, took weeks. Third, the blockchain aspect of the piloted app remains a permanent, institution-agnostic record of the relationship between specific donors and their respective biospecimens, highlighting the potential to reunite individuals with these deeply personal assets, with yet unmet potential for assurances of trust and shared rewards of research. Finally, the biowallet NFT represents a foundational gateway to a composable and progressively decentralized biobanking ecosystem. That which starts with 1 biowallet token per participant who contributes specimens may be built upon in a stepwise manner, forging an interconnected overlay network that recognizes and unlocks value across today’s siloed biobank landscape.

### Limitations

The pilot relied on manual data workflows to enable demonstration of a functional decentralized biobanking platform without requiring full integration of the patient-facing apps with the enterprise system. Such manual workflows are impractical for sustainability and scalability. The exponential growth of health information and advanced computing makes workflow automation increasingly fundamental [37]. Thus, application programming interface (API) integration and automated processes will be necessary for future apps. In view of the volume of requests received during the pilot as well as interest in expanding the program to other institutional biobanks, hospital leadership approved API development to facilitate such integrations for the next stages of the pilot program. In addition to being essential for technical feasibility, this approval was critical as it demonstrated that the manual aspects of our workflows were not material for the acceptability of our strategy for reconnecting donors with their deidentified specimens within institutional biobanks.

Notifications based on in-app activity event triggers were not fully implemented during the pilot, and a number of manual steps were required, including substantial coordination across study team members and email-based messaging to notify participants about critical changes such as token availability and biosample status updates. Automated communications must be incorporated into future pilots with accommodation for a range of patient preferences and values. Subsequent development will also make a web-based version to avoid exclusion of participants for whom smartphone apps may not be preferred or accessible, particularly with respect to age and household income [38].

Furthermore, the piloted app interfaces and user journeys were designed for patient users, whereas engagement with physicians, biobankers, and scientists occurred via alternative channels (eg, email and institutional platforms). This limited the functionality and value within the app as research content was high level, limited to the scope of the biobank database. Ongoing work is advancing real-world applications of decentralized biobanking for scientists and other stakeholders within the NFT digital twin ecosystem. Inclusion of professional users directly within the decentralized biobanking platform will be key for unlocking the ongoing value and network effects of our framework.

Regarding the blockchain elements, the high and highly variable costs of token mints on Ethereum illustrate the importance of more cost-efficient strategies, such as layer-2 solutions, for full-scale implementation. Importantly, our focus on the primary NFT digital twin framework centers the stakeholders and their relational mappings within the ecosystem. This allowed us to focus on tokenizing the individual participants, in this case, 1 token per biospecimen donor rather than 1 per biospecimen, which would have increased costs 10- to 20-fold. This was sensible, especially considering limitations on functionality of a specimen-representing NFT in the setting of our pilot app; that is, it was not necessary to tokenize specimens for implementing transparency and our study did not provide additional permissions relevant to potential tokenized specimen utility for shared governance or profit sharing regarding the underlying biobank assets. Moreover, ensuring the long-term economic sustainability of biobanks is already a salient concern, with high costs driven by human resources, equipment, and sample handling [39-41]. Cost-effectiveness will be essential for broader adoption of decentralized biobanking technology, and blockchain solutions in themselves must be complemented with social, cultural, and legal innovations to enact meaningful progress [40,42,43].

In addition, NFTs were minted for individual participants, and personal NFTs were rendered via an in-app Etherscan display, although the token-gated aspect of the app leveraged Firebase Unique Identifiers rather than NFTs to minimize complexity and potential points of failure. Simulation of the user interface and user experience of blockchain interactions was necessary to overcome barriers to onboarding inherent to contemporary avoidances and constraints of decentralized apps, particularly as our patient population was older and almost exclusively from non–digital native generations and many were actively grappling with cancer. This was especially critical given concurrent educational barriers surrounding the simultaneous introduction of patients to both biobanking and blockchain for the first time. For example, a knowledge assessment on biobanking administered to biospecimen donors found that approximately half of all questions were answered either incorrectly or with “I don’t know.” Similarly, most patients we engaged with during app design, development, and pilot-testing were not familiar with the term “biobank,” illustrating the fundamental challenge of delivering a patient-friendly biobanking app. These findings underscore the gap between providing information during the prospective informed consent process and achieving true comprehension via enduring transparency and ongoing feedback [44,45]. To this end, we prioritized orientation to biobanking and developed lexicon and app design features that make data within biobank databases accessible to donors via a decentralized biobanking platform that coheres with the ethos of decentralization at its core.

For future implementations, we aim to advance blockchain-backed solutions with seamless onboarding experiences through the exploration of newer standards such as ERC-4337 for account abstraction, which awards the programmable flexibility to remove complex barriers to entry such as the current requirement for users to create their own third-party wallets to interact with the decentralized app. Advancement of these technologies may provide seamless integration of decentralized biobanking platforms with both institutional databases and blockchain overlay networks, with future potential to unite participants, specimens, and scientists across various institutions. Transparency and engagement in biospecimen management is a necessary step toward institutional transformation to achieve community partnership, shared decisions, and progressive democratization. More research is needed to test our hypotheses about the role of blockchain technology in a comprehensive and universal decentralized biobanking solution [46].

The success of our pilot inspired potential to revolutionize biobanking via a decentralized platform but also revealed challenges and limitations for current biospecimen collection workflows, standard operating procedures, and data management strategies [47]. Implementation of transparency for past, present, and future biospecimen collection and distribution will require innovative system designs that overcome idiosyncrasies of individual biobank databases coupled with incentive structures and governance models that promote trust and ensure that biobanking practice optimizes individual and collective interests for patients, scientists, and society [48-50]. While the principles and techniques demonstrated in this study theoretically translate to any other research biobanking context, our technical approach must be validated across a variety of clinical and socioeconomic settings, institutional and regional cultures, and biomedical research contexts.

Critically, this pilot addressed a single, disease-focused university biobank with a largely White, female, and geographically localized population. Technology acceptance must be confirmed for diverse patients, diseases, and contexts [51]. Both iOS and Android users were included, yet some did not use smartphones, and others preferred not to download apps. We have since developed a web-based platform, expanding availability to anyone with internet access, although disparities persist. Ongoing research is exploring the impact of age, race, time elapsed since surgery, and stage of disease on technology acceptability, as well as how to optimize recruitment and trustworthiness for underserved populations [51,52]. Current work is also addressing populations such as those with prostate and lung cancer in which male individuals are more heavily represented, and we have incorporated socioeconomic assessments into our data collection to ensure that we advance solutions that are broadly accessible and applicable, especially for economically and educationally marginalized groups.

Looking ahead beyond feasibility, the practical implementation of scalable, decentralized biobanking solutions requires technical enhancements to overcome the discussed challenges and limitations of this pilot. User interfaces must prioritize usability, comprehensibility, and accessibility by leveraging new standards for account abstraction to reduce the complexity of interacting with blockchain components in our solution. Similarly, ongoing research should inform iterative refinement of different strategies for effective presentation of research-related information curated for diverse patient populations. Efforts toward long-term sustainability should include app cost optimization techniques such as deployment on layer-2 networks for major reductions in blockchain transaction costs and the automation of key workflows and processes through proper integration with institutional software and databases. Because each new environment can be quite nuanced, the application of our technology to new use cases will still require custom configurations when onboarding, but some of these efforts may be streamlined by standardizing integration patterns with widely used laboratory information management systems and research tools.

Finally, our privacy-by-design approach requires due diligence in execution to mitigate risks to users. Abiding by security best practices in development and thorough vulnerability testing are essential measures in protecting against critical security risks. Intentional disaster recovery plans with detailed incident response protocols for specific events are important for prompt threat containment, recovery of system resources with minimal downtime, and communication to affected users and stakeholders. Proactive preparation to set up comprehensive monitoring, automated backups with manual snapshots across system resources and environments, and pre-emptively programmed functionality for pausing and redeploying compromised system components or deployed smart contracts are crucial for the effective execution of incident response plans.

### Conclusions

This pilot demonstrates the technical capacity and resources for a functional decentralized biobanking software app that empowers patients to track specimens donated to a real-world breast cancer research biobank with a novel implementation of blockchain technology. The patient-friendly mobile app renders institutional biobank inventory and transactions in a meaningful, personalized biowallet context, providing a rewarding user experience. We demonstrated the app’s readiness for API integrations, which would allow for sustainable and scalable implementation across multiple biobank protocols by seamlessly and dynamically displaying biobanking activities to donors. Pilot participants successfully claimed NFTs within the app, restoring provenance for personal biospecimens and related data. This advancement introduces a new paradigm for ethical biobanking, fostering donor engagement and inclusion in personalized research networks appropriate to contemporary learning health systems and mobile computing capabilities while maintaining deidentification and compliance with established protocols.

## Appendix

Multimedia Appendix 1 Architecture details of the deployed cloud infrastructure, encompassing networking setup, front-end and back-end deployment configurations, database architecture, continuous integration and continuous delivery processes, domain management, and monitoring systems.

Multimedia Appendix 2 Workflow for the de-bi pilot, including consent for the pilot, downloading the app, and minting biowallet tokens to link personal biospecimen data to their biowallet.

Multimedia Appendix 3 Decentralized biobanking pilot study participation rates among eligible biobank members by age, race, and time from initial biobank consent.

Multimedia Appendix 4 Decentralized biobanking pilot study and breast cancer biobank age distributions, and comparison of pilot enrollment rates by time from initial biobank consent.

Multimedia Appendix 5 Demographics of the breast cancer biobank and decentralized biobanking pilot populations.

Multimedia Appendix 6 Decentralized biobanking pilot participant age ranges and engagement metrics among app onboarded participants by sample collection status, including age, biobank membership, years since initial biobank consent, and research profile completion rates.

Multimedia Appendix 7 Characteristics of pilot participants who did versus did not complete research profiles and claim biowallet tokens on decentralized biobanking app.

## Abbreviations

API: application programming interface
BDRR: Breast Disease Research Repository
IRB: institutional review board
NFT: nonfungible token

## Data Availability

The datasets generated or analyzed during this study are available from the corresponding author upon reasonable request.
