# Supplementary material for: Decentralized Biobanking Apps for Patient Tracking of Biospecimen Research: Real-World Usability and Feasibility Study
Source: JMIR Bioinform Biotechnol. 2025 Apr 10;6:e70463. doi: 10.2196/70463 (PMC12022527; doi:10.2196/70463)
Supplement: Multimedia Appendix 1 [file bioinform_v6i1e70463_app1.docx]

**Multimedia Appendix 1.** Architecture details of the deployed cloud infrastructure, encompassing networking setup, frontend and backend deployment configurations, database architecture, CI/CD processes, domain management, and monitoring systems.

| **Multimedia Appendix Table 1. Cloud Architecture Details** |
| --- |
| Deployed Cloud Infrastructure includes, but is not limited to, the following:  **Networking**   - Creation of a Virtual Private Cloud - Creation of Public and Private Subnets - Creation of Internet Gateways - Creation of NAT Gateways - Creation of Security Groups - Creation of Bastion Host   **Frontend & Backend Deployment**   - Network Allocation on the VPC and Availability Zones, Setting up the Load Balancer on a Public subnet and the EC2 instance on a private subnet - Port redirection from HTTP to HTTPS - Creation of an ACM Certificate to enable the SSL endpoint - Compilation and hooks for the code to be deployed on the Beanstalk - Elastic Beanstalk Environment Setup   **Database**   - Creation of the Aurora DB cluster and DB Instance. The database is only private accessible from the resources inside the VPC and from the bastion host. Database Encryption is enabled. - The database Credentials are stored in AWS Secrets Manager.   **CI/CD**   - Setting up Continuous Integration and Continuous Deployment to detect, build and deploy the latest changes on the Github repositories to the Elastic Beanstalk Environments - Integration of the Github repos with AWS - CodePipeline - CodeBuild component to Build applications code in case it’s necessary.   **Domain Management**   - Transfer the management of the Domain from GoDaddy to Route53 - Creation of a Hosted Zone in Route53 - Creation of Records Inside the Hosted Zone   **Monitoring**  Each one of the components of the architecture and the AWS account needs to be monitored to track performance, detect issues, audit, and troubleshoot.   - CloudWatch Logs group to store application logs. - CloudWatch Dashboards to monitor application’s performance. - CloudWatch Alarms will trigger notifications in case an event of interest is detected. - CloudTrail to audit and keep track of all the changes made in the AWS account related to the project. |
