# Supplementary material for: Decentralized Biobanking Apps for Patient Tracking of Biospecimen Research: Real-World Usability and Feasibility Study
Source: JMIR Bioinform Biotechnol. 2025 Apr 10;6:e70463. doi: 10.2196/70463 (PMC12022527; doi:10.2196/70463)

**Multimedia Appendix 2: Overview of de-bi pilot activities**

Workflow for the de-bi pilot, including consent for the pilot, downloading the app, and minting biowallet tokens to link personal biospecimen data to their biowallet.


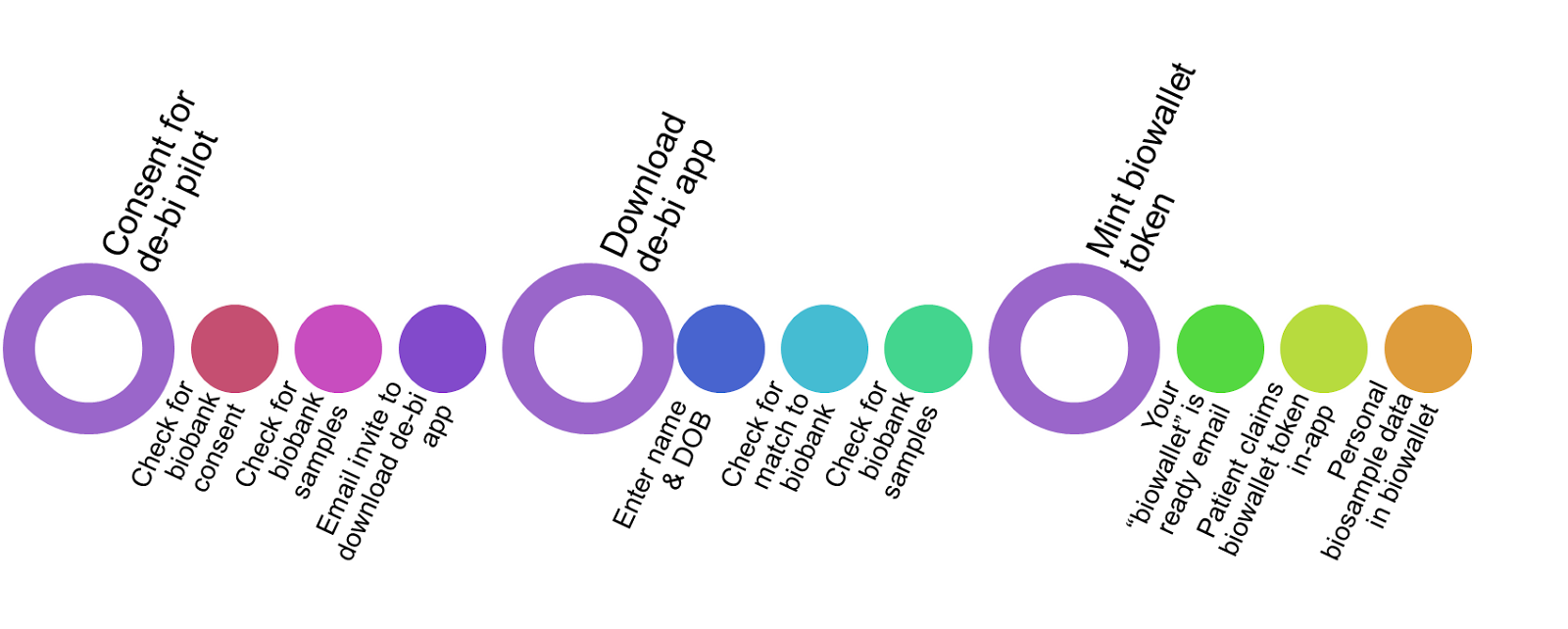

Supplement: Multimedia Appendix 2 [file bioinform_v6i1e70463_app2.docx]
