# Supplementary material for: Decentralized Biobanking Apps for Patient Tracking of Biospecimen Research: Real-World Usability and Feasibility Study
Source: JMIR Bioinform Biotechnol. 2025 Apr 10;6:e70463. doi: 10.2196/70463 (PMC12022527; doi:10.2196/70463)
Supplement: Multimedia Appendix 3 [file bioinform_v6i1e70463_app3.docx]

**Multimedia Appendix 3.** Decentralized biobanking pilot study participation rates among eligible biobank members by age, race, and time from initial biobank consent.

|  | Pilot consented (N)* | Biobank members eligible for pilot (N)* | Proportion pilot consented (%) | SE (%) | P¹P_0_ |
| --- | --- | --- | --- | --- | --- |
| Total (P_0_) | 930 | 9750 | 9.5 | 0.3 |  |
| Age | | | | | |
| Under 40 | 78 | 466 | 16.7 | 1.7 | **<0.001** |
| 40-49 | 163 | 883 | 18.5 | 1.3 | **<0.001** |
| 50-59 | 242 | 1924 | 12.6 | 0.8 | **<0.001** |
| 60-69 | 247 | 2808 | 8.8 | 0.5 | 0.199 |
| 70+ | 197 | 3668 | 5.4 | 0.4 | **<0.001** |
| Biobank Consent | | | | | |
| During Pilot | 32 | 261 | 12.3 | 2.0 | 0.134 |
| <1 Years | 161 | 1056 | 15.3 | 1.1 | **<0.001** |
| 1-2 Years | 111 | 945 | 11.8 | 1.0 | **0.021** |
| 2-3 Years | 84 | 646 | 12.4 | 1.3 | **0.011** |
| 3-4 Years | 70 | 682 | 10.3 | 1.2 | 0.519 |
| 4-5 Years | 79 | 939 | 8.4 | 0.9 | 0.240 |
| 5-10 Years | 272 | 2912 | 9.3 | 0.5 | 0.716 |
| 10-15 Years | 81 | 1144 | 7.1 | 0.8 | **0.005** |
| >15 Years | 31 | 533 | 5.8 | 1.0 | **0.003** |
| Race | | | | | |
| White | 884 | 8823 | 10.0 | 0.3 | 0.101 |
| Black | 29 | 676 | 4.3 | 0.3 | **<0.001** |
| Asian | 7 | 118 | 5.9 | 2.2 | 0.186 |
| Native American or Alaska Native** | 0 | 17 | 0 | 0 | 0.183 |

SE (Standard error of sample proportion)

P_0_ (Overall biobank population rate of de-bi pilot participation)

P≠P_0_ (de-bi participation rate among demographic sub-population is not equal to the overall biobank population de-bi pilot participation rate)

< (Less than)

> (Greater than)

*Population demographic proportions were calculated from data with parameters of interest known. Not all entries in biobank and pilot data sets had age data available. Rate of decentralized biobanking pilot consent during study was calculated based on consent data collected during active pilot recruitment (February 16, 2023 – May 4, 2023). Only pilot participants who were biobank members had race and date of initial biobank consent data available.

**One-sided binomial exact test
