# Supplementary material for: Decentralized Biobanking Apps for Patient Tracking of Biospecimen Research: Real-World Usability and Feasibility Study
Source: JMIR Bioinform Biotechnol. 2025 Apr 10;6:e70463. doi: 10.2196/70463 (PMC12022527; doi:10.2196/70463)
Supplement: Multimedia Appendix 4 [file bioinform_v6i1e70463_app4.docx]

**Multimedia Appendix 4.**  Decentralized biobanking pilot study and breast cancer biobank age distributions, and comparison of pilot enrollment rates by time from initial biobank consent.


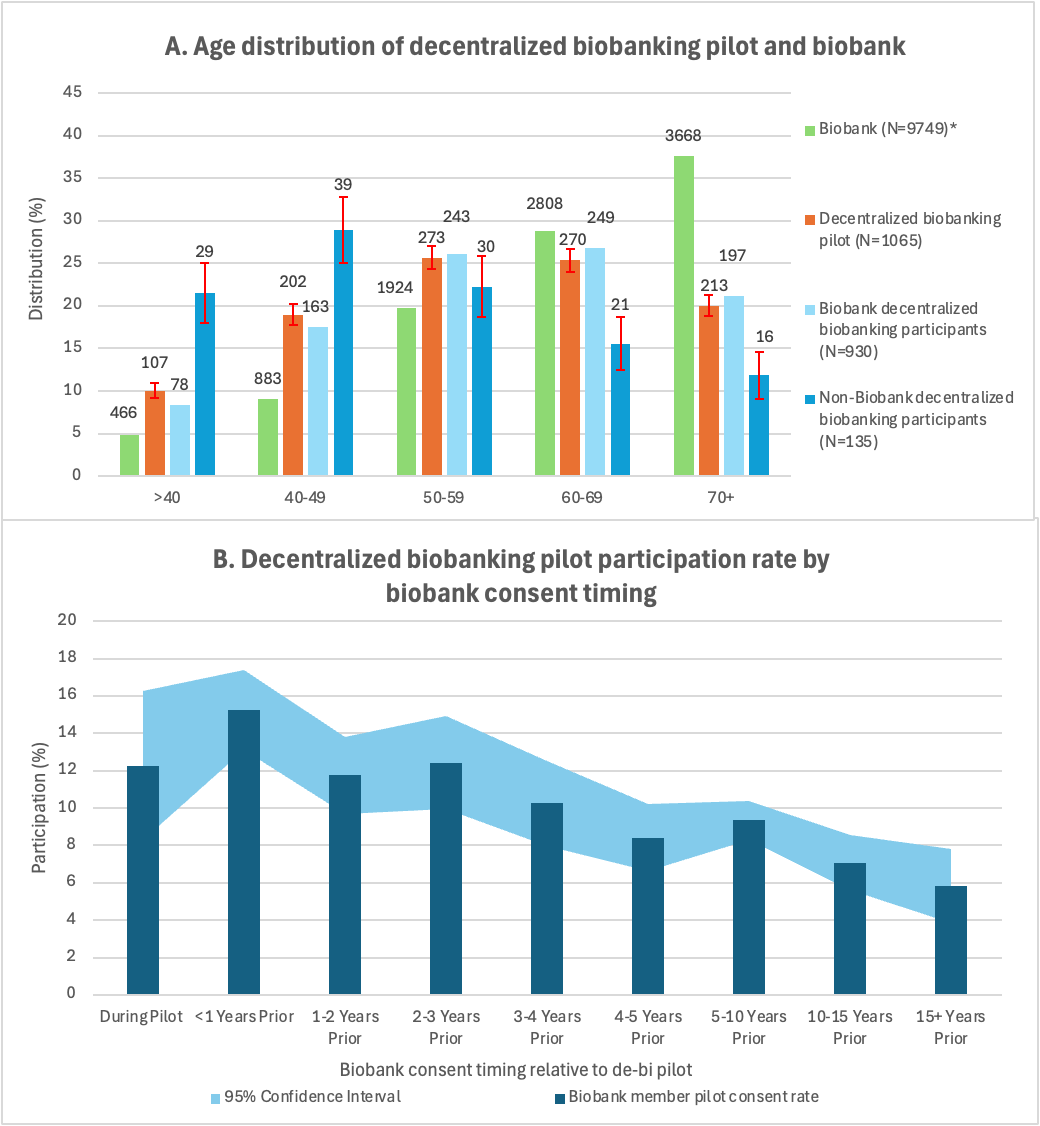


| A. Histogram illustrating variability in age distributions of four populations: biobank (green) and decentralized biobanking pilot (orange) populations overall, as well as biobank consented (light blue) and non-biobank consented decentralized biobanking participants (dark blue). Each bar represents the proportion of individuals comprising each age group from the respective population, highlighting notable differences in age demographics. Age composition of the pilot population overall was skewed younger than the overall biobank population. Of pilot participants, non-biobank consented participants were skewed younger than those who were biobank consented. Population age distributions were calculated from entries with age data available.  B. Bar graph demonstrating consent rates of biobank members to decentralized biobanking pilot by time in years from biobank consent to beginning of decentralized biobanking study (2/16/23). Individuals considered biobank consented during study consented to the biobank during active recruitment (February 16, 2023 - May 4, 2023). Higher pilot consent rates than the overall population rate (9.5%; 930/9749), were observed in individuals consented to the biobank closer to de-bi pilot launch (< 3 years prior: p <.001). Pilot consent rates from the breast cancer biobanks were calculated from entries where initial biobank consent date data were available. |
| --- |
