# Supplementary material for: Decentralized Biobanking Apps for Patient Tracking of Biospecimen Research: Real-World Usability and Feasibility Study
Source: JMIR Bioinform Biotechnol. 2025 Apr 10;6:e70463. doi: 10.2196/70463 (PMC12022527; doi:10.2196/70463)
Supplement: Multimedia Appendix 5 [file bioinform_v6i1e70463_app5.docx]

**Multimedia Appendix 5.** Demographics of the breast cancer biobank and decentralized biobanking pilot populations.

| **Decentralized biobanking pilot and biobank population demographics** | | | | | | | | | | | | | | | | | | | | | | | | | | | | | | | | | | | | | |
| --- | --- | --- | --- | --- | --- | --- | --- | --- | --- | --- | --- | --- | --- | --- | --- | --- | --- | --- | --- | --- | --- | --- | --- | --- | --- | --- | --- | --- | --- | --- | --- | --- | --- | --- | --- | --- | --- |
| Pilot (N=1080) | | | | | | | | | | | | | | | | | | | | | | | | | | | | Biobank (N=9750)** | | | | | | | | | |
| Biobank members | | Not biobank members | | | | | Specimen data available | | | | | | Specimens collected | | | | | | | Specimen distributed for research | | | | | | | | In pilot | | | | | | Not pilot participants | | | |
| 930 (86.1) | | 150 (13.9) | | | | | 926 (85.7) | | | | | | 272 (25.2) | | | | | | | 174 (16.1) | | | | | | | | 930 (9.5) | | | | | | 8820 (90.5) | | | |
| **Mean Age Comparisons (Mean (SD))** | | | | | | | | | | | | | | | | | | | | | | | | | | | | | | | | | | | | | |
| Pilot | Biobank | | Pilot ¹ Biobank | | | Pilot + Biobank | | | | | | Pilot not in biobank | | | Biobank ¹ Not Biobank | | | | Biobank not in pilot | | | | Pilot + Biobank ¹ Biobank not in Pilot | | | | 1st Pilot email  (N=260) | | | | Last Pilot email (N=237) | | | | | | 1st Week ¹ Last Week |
| 57.2 (13.5) | 64.3 (13.6) | | **<.001** | | | 58.1 (13.1) | | | | | | 50.5 (13.8) | | | **<.001** | | | | 64.9 (13.5) | | | | **<.001** | | | | 55.1 (12.8) | | | | 60.1 (13.4) | | | | | | **<.001** |
| **Distribution of decentralized biobanking pilot vs. overall biobank populations** | | | | | | | | | | | | | | | | | | | | | | | | | | | | | | | | | | | | | |
| **Age** | | **N (%)** | | | | | | | | | | | | | | | | | | | | | | | | | | | | | | | | | | | |
|  |  | <40 | | | | | | | 40-49 | | | | | | | | 50-59 | | | | | | | | 60-69 | | | | | | | | 70+ | | | | |
| Pilot* (N=1065) | | 107 (10.1) | | | | | | | 202 (19.0) | | | | | | | | 273 (25.6) | | | | | | | | 270 (25.4) | | | | | | | | 213 (20.0) | | | | |
| Biobank (N=9749) | | 466 (4.8) | | | | | | | 883 (9.1) | | | | | | | | 1924 (19.7) | | | | | | | | 2808 (28.8) | | | | | | | | 3668 (37.6) | | | | |
| Pilot ¹ Biobank | | **<.001** | | | | | | | **<.001** | | | | | | | | **<.001** | | | | | | | | 0.178 | | | | | | | | **<.001** | | | | |
|  | | | | | | | | | | | | | | | | | | | | | | | | | | | | | | | | | | | | | |
| **Race** | | White | | | | | | | | | Black or African American | | | | | | | | | Asian | | | | | | | | | Native American or Alaska Native | | | | | | | | |
| Pilot + Biobank (N=920) | | 884 (96.1) | | | | | | | | | 29 (0.6) | | | | | | | | | 7 (0.8) | | | | | | | | | 0 (0) | | | | | | | | |
| Biobank (N=9634) | | 8822 (91.6) | | | | | | | | | 676 (7.0) | | | | | | | | | 118 (1.2) | | | | | | | | | 17 (0.2) | | | | | | | | |
| Pilot + Biobank ¹ Biobank | | **< .001** | | | | | | | | | **< .001** | | | | | | | | | 0.215 | | | | | | | | | 0.201 | | | | | | | | |
|  | | | | | | | | | | | | | | | | | | | | | | | | | | | | | | | | | | | | | |
| **Biobank Consent Timing** | | During Pilot | | | <1 Years Prior | | | | | 1-2 Years Prior | | | | 2-3 Years Prior | | | | 3-4 Years Prior | | | | 4-5 Years Prior | | | | 5-10 Years Prior | | | | 10-15 Years Prior | | | | | | >15 Years Prior | |
| de-bi Pilot (N=926) | | 37 (4.0) | | | 161 (17.4) | | | | | 111 (12.0) | | | | 84 (9.1) | | | | 70 (7.6) | | | | 79 (8.6) | | | | 272 (29.4) | | | | 81 (8.7) | | | | | | 31 (3.3) | |
| Biobank (N=9744) | | 857 (8.8) | | | 1056 (10.8) | | | | | 945 (9.7) | | | | 676 (6.9) | | | | 682 (7.0) | | | | 939 (9.6) | | | | 2912 (29.9) | | | | 1144 (11.7) | | | | | | 533 (5.5) | |
| Pilot ¹ Biobank | | **<.001** | | | **<.001** | | | | | **0.026** | | | | **0.0159** | | | | 0.524 | | | | 0.274 | | | | 0.745 | | | | **0.006** | | | | | | **0.006** | |
| **App Engagement** | | | | | | | | | | | | | | | | | | | | | | | | | | | | | | | | | | | | | |
| # App Engaged | | | | | | | | App Engaged Specimen ¹ No Specimen | | | | | | | | Age App Engaged (Mean (SD)) | | | | | | | | Age Not App Engaged (Mean (SD)) | | | | | | | | Age App Engaged ¹ Age Not Engaged | | | | | |
| Pilot (N=1080) | | | | 405 (37.5) | | | |  |  |  |  |  |  |  |  |  |  |  |  |  |  |  |  |  |  |  |  |  |  |  |  |  |  |  |  |  |  |
| No specimen (N=808) | | | | 257 (31.8) | | | | **< .001** | | | | | | | | 55.6 (12.8) | | | | | | | | 58.2 (13.8) | | | | | | | | **0.002** | | | | | |
| Specimens (N=272) | | | | 148 (54.4) | | | |  |  |  |  |  |  |  |  |  |  |  |  |  |  |  |  |  |  |  |  |  |  |  |  |  |  |  |  |  |  |
| **Pilot Participants with Biospecimens** | | | | | | | | | | | | | | | | | | | | | | | | | | | | | | | | | | | | | |
| Pilot specimen data available (N=926) | | | | | | | | Age | | | | | | | Number of specimens per participant | | | | | | | | | | | | | Number specimens distributed per participant | | | | | | | | | |
|  |  |  |  |  |  |  |  | Mean (SD) | | | | | | | Specimens total | | | | | | Mean (SD) | | | | | | | Specimens total | | | | | | | Mean (SD) | | |
| Pilot with specimens | | | | 272 (29.4) | | | | 60.8 (12.0) | | | | | | | 3912 | | | | | | 14.4 (11.8) | | | | | | | 596 | | | | | | | 3.4 (6.6) | | |
| App user with specimens | | | | 148 | | | | 58.5 (11.4) | | | | | | | 2133 | | | | | | 14.4 (12.1) | | | | | | | 376 | | | | | | | 4.0 (8.0) | | |
| App users with specimens in Use | | | | 94 | | | |  |  |  |  |  |  |  |  |  |  |  |  |  |  |  |  |  |  |  |  |  |  |  |  |  |  |  |  |  |  |

≠ (Not equal to)

SD (Standard Deviation)

< (Less than)

> (Greater than)

*Population demographic proportions were calculated from data with parameters of interest known. Not all entries in biobank, and decentralized biobanking pilot datasets had age data available. Only pilot participants who were biobank members had race and date of initial biobank consent known. Biospecimen data was only available for biobank members, and some individuals may not have matched to their respective biospecimens due participant typos on the electronic de-bi enrollment consent form and/or institutional database errors that were discovered during data analysis

**Pilot enrollment rates calculated from recruited biobank members who were living at the beginning of the pilot study (January 1, 2023).
