# Supplementary material for: Decentralized Biobanking Apps for Patient Tracking of Biospecimen Research: Real-World Usability and Feasibility Study
Source: JMIR Bioinform Biotechnol. 2025 Apr 10;6:e70463. doi: 10.2196/70463 (PMC12022527; doi:10.2196/70463)
Supplement: Multimedia Appendix 6 [file bioinform_v6i1e70463_app6.docx]

**Multimedia Appendix 6.** Decentralized biobanking pilot participant age ranges and engagement metrics among app onboarded participants by sample collection status, including age, biobank membership, years since initial biobank consent, and research profile completion rates.

| Decentralized Biobanking App Engagement by Sample Collection Status | | | |
| --- | --- | --- | --- |
|  | Samples collected (N=148) | No Samples collected (N=257) | All app users  (N=405) |
| Age (Min-Max) | 57.5 (11-85) | 53.2 (21-86) | 54.7 (11-86) |
| Biobank Members(N=213) |  | 54.0 (21-79) |  |
| Non-Biobank Members  (N=44) |  | 49.1 (21-86) |  |
| Years Since Biobank Consent (Min-Max) | 7.8 Years  (25 Days - 16.9 Years) | 3.6 Years  (69 Days Prior - 16.7 Years) | 5.3 Years  (69 Days Prior – 16.9 Years) |
|  | | | |
| Completed One or More Research Profile Sections | 59 (39.60%) | 94 (36.58%) | 153 (37.78%) |
| Age (Min-Max) | 58.7 (39-81) | 52.6 (21-79) | 54.9 (21-81) |
| Biobank Members (N=75) |  | 53.1 (21-79) |  |
| Non-Biobank Members (N=19) |  | 50.7 (21-73) |  |
| Years Since Biobank Consent (Min-Max) | 8.3 Years  (70 Days – 16.6 Years) | 3.5 Years  (69 Days Prior – 14.4 Years) | 5.6 Years  (69 Days Prior – 16.6 Years) |
|  | | | |
| Did not Complete Research Profile | 89 (60.14%) | 138 (53.70%) | 252 (62.22%) |
| Age (Min-Max) | 56.7 (11-85) | 53.5 (21-86) | 54.6 (11-86) |
| Biobank Members (N=138) |  | 54.5 (21-77) |  |
| Non-Biobank Members (N=25) |  | 48.0 (24-86) |  |
| Years Since Biobank Consent (Min-Max) | 7.5 Years  (25 Days – 16.9 Years) | 3.6 Years  (19 Days – 16.7 Years) | 5.1 Years  (19 Days – 16.9 Years) |
