# Supplementary material for: Decentralized Biobanking Apps for Patient Tracking of Biospecimen Research: Real-World Usability and Feasibility Study
Source: JMIR Bioinform Biotechnol. 2025 Apr 10;6:e70463. doi: 10.2196/70463 (PMC12022527; doi:10.2196/70463)
Supplement: Multimedia Appendix 7 [file bioinform_v6i1e70463_app7.docx]

**Multimedia Appendix 7.** Characteristics of pilot participants who did versus did not complete research profiles and claim biowallet tokens on decentralized biobanking app.

**Research profile completion:** The average age of those who did not fill out the research profile is 56.3 years (SD 12.5 years; range = 18 to 87.4 years), while those who did fill out the profile have a slightly higher average age of 56.6 years (SD 13.6 years; range = 22.3 to 82.2 years). In terms of time since consent, those who did not fill out the profile have an average time since BDRR consent of 5.2 years (SD 4.3 years; range = 0.1 to 16.9 years), compared to 5.6 years (SD 4.7 years; range = -0.2 to 16.6 years) for those who did. The proportion of individuals in the biobank is 90.1% (227/252) for those who did not fill out the profile and 87.6% (133/153) for those who did. Finally, 35.3% (89/252) of individuals who did not fill out the profile are biobank members, compared to 38.6% (59/153) of those who did.

**Biowallet claiming:** Comparison of individuals who claimed their biowallet versus those who did not reveals that those who did tend to be slightly younger and have a slightly higher participation rate in BDRR and as BDRR donors compared to those who did not claim their biowallet. Specifically, individuals who did not claim their biowallet have an average age of 61.9 years (SD 14.3 years; range = 37.05 to 87.37 years), whereas those who claimed their biowallet have a slightly lower average age of 58.91 years (SD 10.8 years; range = 39.16 to 80.95 years). Regarding the time since BDRR consent, those who did not claim their biowallet have an average of 7.7 years since consent (SD 5.28 years; range = 1.56 to 16.46 years), while those who claimed their biowallet have a similar average of 7.75 years since consent (4.95; range = 0.07 to 16.90 years). When looking at biobank membership status, all individuals who did not claim their biowallet are in the biobank (100%; 17/17), compared to 98.5% (128/130) of those who claimed their biowallet. Finally, among BDRR donors, 88% (15/17) of individuals who did not claim their biowallet are BDRR donors, compared to 96.2% (125/130) of those who claimed their biowallet.
